# A strategy to design protein-based antagonists against type I cytokine receptors

**DOI:** 10.1371/journal.pbio.3002883

**Published:** 2024-11-26

**Authors:** Timo Ullrich, Olga Klimenkova, Christoph Pollmann, Asma Lasram, Valeriia Hatskovska, Kateryna Maksymenko, Matej Milijaš-Jotić, Lukas Schenk, Claudia Lengerke, Marcus D. Hartmann, Jacob Piehler, Julia Skokowa, Mohammad ElGamacy

**Affiliations:** 1 Max Planck Institute for Biology, Department of Protein Evolution, Tübingen, Germany; 2 Translational Oncology, Internal Medicine II, University Hospital Tübingen, Tübingen, Germany; 3 Department of Biology/Chemistry and Center for Cellular Nanoanalytics, Osnabrück University, Osnabrück, Germany; 4 Interfaculty Institute of Biochemistry, University of Tübingen, Tübingen, Germany; Stanford University, UNITED STATES OF AMERICA

## Abstract

Excessive cytokine signaling resulting from dysregulation of a cytokine or its receptor can be a main driver of cancer, autoimmune, or hematopoietic disorders. Here, we leverage protein design to create tailored cytokine receptor blockers with idealized properties. Specifically, we aimed to tackle the granulocyte-colony stimulating factor receptor (G-CSFR), a mediator of different types of leukemia and autoinflammatory diseases. By modifying designed G-CSFR binders, we engineered hyper-stable proteins that function as nanomolar signaling antagonists. X-ray crystallography showed atomic-level agreement with the experimental structure of an exemplary design. Furthermore, the most potent design blocks G-CSFR in acute myeloid leukemia cells and primary human hematopoietic stem cells. Thus, the resulting designs can be used for inhibiting or homing to G-CSFR-expressing cells. Our results also demonstrate that similarly designed cytokine mimics can be used to derive antagonists to tackle other type I cytokine receptors.

## Abstract 2

Protein-based therapies have been developed to block cytokine signaling in a range of different diseases. These authors use a computational protein design approach to develop novel cytokine receptor antagonists and show that the designed proteins can inhibit G-CSFR activation in a cell model of acute myeloid leukemia.

## Introduction

Inhibiting cytokine signaling can be highly effective in treating various cancers, autoimmune, and hematological disorders. Highly specific protein-based therapies have been developed to block cytokine signaling to tackle a range of diseases [1]. These include cytokine-blocking antibodies [2,3] and recombinant soluble receptors [4,5], or alternatively, receptor-blocking antibodies [6,7] and cytokine-based antagonists [8,9]. Computational protein design unlocks new strategies for creating purpose-tailored inhibitors with optimal structural, biophysical, and functional features [10,11]. Here, we describe an approach for antagonizing cytokine receptor signaling based on designed cytokine mimics with idealized structural and biophysical properties. We use this approach with the aim of blocking the granulocyte-colony stimulating factor receptor (G-CSFR) signaling, a crucial driver of granulopoiesis, that when dysregulated can promote tumor progression and inflammatory diseases [12].

G-CSFR is activated through ligand-induced dimerization of the ectodomains of 2 receptor subunits, which leads to the cross-phosphorylation and activation of the intracellular receptor-associated kinases [13]. Physiologically, G-CSFR signaling is primarily responsible for the proliferation and differentiation of hematopoietic stem cells into neutrophils, and for mobilization of hematopoietic cells and neutrophils into peripheral blood from the bone marrow [14]. Additionally, it has pro-proliferative and pro-survival effects on neuronal, vascular, and myocardial tissues [15–18]. Conversely, G-CSFR signaling promotes several types of leukemia [13,19,20] and solid tumors [21–25]. It also constitutes a driver of inflammatory disorders [26,27], including rheumatoid arthritis [28–30], further motivating the development of specific G-CSFR inhibitors.

The native ligand (G-CSF) engages and dimerizes G-CSFR through 2 binding sites in a 2:2 receptor:ligand complex that juxtaposes the receptor subunits into a signaling-competent configuration. Through this study, we aimed to design a series of bifaceted, designed ligands which could engage G-CSFR either in a non-signaling ternary complex, or in a 1:1 stoichiometry, outcompeting native G-CSF. We follow this up by a detailed biophysical and functional characterization to delineate the inhibitory potential of the most promising designs. We further carry out computationally guided functional enhancement and demonstrate the capacity of our designs to antagonize G-CSF in acute myeloid leukemia and primary hematopoietic stem cells. The approach of designing small binding proteins with a simplified fold is superior to previously described strategies and can be readily extended to tackle other target cytokine and growth factor receptors.

## Materials and methods

### Ethics statement

All experiments in this study involving human-derived HSCPs samples were conducted according to Helsinki’s declaration, and study approval was obtained from the Ethical Review Board of the Medical Faculty, University of Tübingen, under study approval number 725/2022BO2.

### Modeling of bifaceted variants of G-CSFR-binding designs

The original Boskar4 template encodes a single G-CSFR-binding site (PDB: 7NY0 [31]) that matches site II of the G-CSF (PDB: 2D9Q [32]). This binding site is mounted across the helix 2 and helix 3 (H2/3) regions of the Boskar4 starting template, herein referred to as the primary binding site. Boskar4 structure however allows for grafting another binding site on helices 1 and 4 (H1/H4) due to the pseudosymmetry of the 4-helix-bundle, which can constitute a secondary binding site. This additional graft of a secondary binding site on H1/H4 was performed in both parallel and antiparallel orientations with respect to the primary binding site, where an AlphaFold2 [33] model of the affinity-enhanced variant of Boskar4; bv6 [34] was used. The antiparallel grafts, named *boa* (bivalent-orientation antiparallel) were modeled using either a selection of 12 or 15 binding-site residues, denoted as boa1 and boa2, respectively (Table A in S1 Text). The parallel orientation grafts, named *bop* (bivalent-orientation parallel), were modeled using either 12 or 17 binding-site residues, denoted as bop1 and bop2, respectively (Table A in S1 Text). The grafting position was adjusted to minimize the backbone alignment RMSD of the re-grafted site. The sequences of the final designs are provided in Table B in S1 Text. The ternary complexes of the resulting design models were modeled using AlphaFold3 server [35].

### Protein overexpression and purification

Synthetic genes of all designs were cloned into a pET28a downstream of N-terminal His-tag and thrombin cleavage site. Expression was performed in *Escherichia coli* (*E*. *coli*) BL21(DE3) cells grown in LB medium supplemented with 40 μg/ml kanamycin in a shaking flask incubator 37 °C and 160 rpm. Cells were induced at an optical density at 600 nm (OD_600_) of approximately 0.6, using 0.5 mM IPTG, and the expression of the target protein was carried out at 25 °C and 160 rpm for approximately 16 h. After 16 h, the cells were harvested by centrifugation at 5,000 *g* for 30 min. The supernatant was discarded, and the cell pellet was lysed using ultrasonic homogenization in a lysis buffer containing 50 mM Tris (pH 8), 100 mM NaCl, 20 μg/ml DNaseI (A3778, ITW Reagents), and a protease inhibitor cocktail (04693132001, Roche). The lysate was then centrifugated at 16,000 *g* for 30 min, and the resulting supernatant was loaded onto nickel immobilized metal affinity chromatography (IMAC) beads for batch purification (11912422, Macherey-Nagel). Subsequently, size exclusion chromatography (SEC) was performed on a Superdex 75 Increase 10/300 GL (29148721, Cytiva) using PBS as the mobile phase. Fractions expected to contain the protein of the desired size were concentrated using ultrafiltration with a 10 kDa cutoff membrane (UFC8010, Amicon Ultra) and were analyzed again with SEC under the same conditions. Aliquots of these concentrated fractions were frozen at −20 °C until further use.

### Analysis of folding stability

Circular dichroism (CD) spectra were obtained using a JASCO J-810 spectrometer for protein samples diluted to a concentration of 0.2 mg/ml in PBS buffer at pH 7.0. The CD spectra were recorded by measuring the residual ellipticity of 3 accumulations, with a resolution of 0.1 nm over a range of 250 to 190 nm. To determine the melting temperature with CD, the average residual ellipticity was measured at a wavelength of 222 nm over a temperature range of 20 °C to 100 °C with a temperature increase of 0.5 °C per minute. Nanoscale-differential scanning fluorimetry (nanoDSF) experiments were carried out using a Prometheus NT.48 instrument (Nanotemper Technologies) in standard Prometheus capillaries (Nanotemper, PR-C002). These measurements were made on protein samples with a concentration of 1 mg/ml in PBS. The temperature ramp for melting and cooling was set at 1 °C per min, ramping from 25 °C to 110 °C, followed by a reverse ramp back to 25 °C.

### Binding affinity determination using surface plasmon resonance

To evaluate the affinity of the designs to G-CSFR, multi-cycle kinetics experiments were conducted using a Biacore X100 system (GE Healthcare Life Sciences). Recombinant human G-CSFR (Glu25-Pro621, 381-GR/CF, R&D Systems) was diluted to a concentration of 50 μg/ml in a 10 mM acetate buffer at pH 5.0 and immobilized on a CM5 sensor chip (GE Healthcare 29149604) using amine coupling chemistry. The protein samples were diluted in a running buffer consisting of PBS with 0.05% v/v Tween-20. The experiments were carried out at a temperature of 25 °C and a flow rate of 30 μl/min. The sample solutions were injected sequentially over the functionalized sensor chip surface for 180 s, followed by a dissociation phase of 600 s with running buffer. Four different concentrations for each sample were measured which are indicated in the corresponding legend of the corresponding figure. After each run, the sensor surface was regenerated by injecting a 50 mM NaOH solution for 60 s. To analyze the data, the reference responses and zero-concentration sensograms were subtracted from each data set (double-referencing). The Biacore X100 Evaluation Software following a 1:1 binding kinetic model was used to determine the association rate (*k*_*a*_), dissociation rate (*k*_*d*_), and equilibrium dissociation constant (*K*_*D*_) constants. Repeated leave-one-out fitting was performed to determine mean and standard deviation of *k*_*a*_, *k*_*d*_, and *K*_*D*_.

### Single-molecule imaging in live cells

For cell surface labeling, G-CSFR lacking the signal sequence was N-terminally fused to the ALFA-tag (ALFAtag-G-CSFR) [36] and cloned into a pSems vector that included the signal sequence of Igκ (pSems-leader) [37]. JAK2 lacking the tyrosine kinase domain (JAK2ΔTK) C-terminally fused to mEGFP (JAK2ΔTK-mEGFP) cloned into the pSems vector was co-transfected to enable JAK-mediated stabilization of receptor dimers [38]. HeLa cells (ACC 57, DSMZ Germany) were cultured as previously described [37]. For transient transfection, cells were incubated for 4 to 6 h with a mixture of 150 mM NaCl, 10 μl of 1 mg/ml polyethylenimine (PEI MAX, Polysciences 24765) and 1,000 ng (ALFAtag-G-CSFR) and 2,500 ng (JAK2ΔTK-mEGFP) of the desired constructs. Labeling, washing, and subsequent imaging were performed after mounting the coverslips into custom-made incubation chambers with a volume of 1 ml [39]. Cells were equilibrated in medium with FBS but lacking phenol red and supplemented with an oxygen scavenger and a redox-active photoprotectant (0.5 mg/ml glucose oxidase (Sigma-Aldrich), 0.04 mg/ml catalase (Roche), 5% w/v glucose, 1 μm ascorbic acid, and 1 μm methylviologene) to minimize photobleaching [40].

Selective cell surface receptor labeling was achieved by using anti-ALFAtag NBs, which were site-specifically labeled by maleimide chemistry via a single cysteine residue at their C-termini [40]. NBs labeled with Cy3B (DOL: 1.0) and ATTO 643 (DOL: 1.0) were added at concentrations of 3 nM each, at least 10 min before imaging. Coverslips were precoated with poly-l-lysine-graft-poly(ethylene glycol) to minimize unspecific binding of NBs and functionalized with RGD peptide for efficient cell adhesion [41].

Single-molecule imaging was carried out by dual-color total internal reflection fluorescence (TIRF) microscopy using an inverted microscope (IX71, Olympus) equipped with a spectral image splitter (DualView, Optical Insight) and a back-illuminated electron-multiplied CCD camera (iXon DU897D, Andor Technology). Fluorophores were excited by simultaneous illumination with a 561-nm laser (CrystaLaser; approximately 32 W cm^−2^) and a 642-nm laser (Omicron: approximately 22 W cm^−2^). Image stacks of 150 frames were recorded for each cell at a time resolution of 32 ms per frame, with at least 10 cells recorded in each experiment. Ligands were incubated for 10 min before imaging. All imaging experiments were carried out at room temperature (RT). Prior to image acquisition, the presence of JAK2ΔTK-mEGFP was confirmed through emission with a 488-nm laser (Sapphire LP, Coherent).

Dual-color time-lapse images were evaluated using an in-house developed MATLAB software (SLIMfast4C, https://zenodo.org/record/5712332) as previously described in detail [40]. After channel registration based on calibration with fiducial markers, molecules were localized using the multi-target tracking algorithm [42]. Immobile emitters were filtered out by spatiotemporal cluster analysis [43]. For co-tracking, frame-by-frame co-localization within a cutoff radius of 100 nm was applied followed by tracking of co-localized emitters using the utrack algorithm [44]. Molecules co-diffusing for 10 frames or more were identified as co-localized. Relative levels of co-localization were determined based on the fraction of co-localized particles as described elsewhere in detail [45,46]. Diffusion properties were determined from pooled single trajectories using mean squared displacement analysis for all trajectories with a lifetime greater than 10 frames. Diffusion constants were determined from the mean squared displacement by linear regression.

### NFS-60 cell proliferation assay

The NFS-60 cells [47,48] were cultured at 5% CO_2_ and 37 °C in NFS-60-medium, which consisted of RPMI 1640 medium supplemented with 1 mM L-glutamine, 1 mM Na-pyruvate, 10% FCS, 1% Antibiotic-Antimycotic (15240062, Gibco), and 12.5% KMG-2: 5637-CM (Conditioned Medium from CLS Cell Lines Service). Prior to the proliferation assay, the cells were washed 3 times with NFS-60-medium without KMG-2. The assay was conducted in black 96-well plates. Each well contained 45,000 cells in a total volume of 150 μl NFS-60-medium without KMG-2, cultured under maintenance conditions with different concentrations of the protein sample for 48 h. Following that, 30 μl of CellTiter-Blue Reagent (G808, Promega) was added to each well, and the cells were further incubated for approximately 90 min at 5% CO_2_ and 37 °C. Subsequently, the fluorescence of each well was measured on a plate reader with an excitation wavelength of 560 nm and emission at 590 nm. The half-maximal effective concentration (*EC*_*50*_) of each protein sample was determined by fitting the obtained fluorescence values to their corresponding concentrations using a four-parameter sigmoidal function minimized with the Nelder-Mead Simplex algorithm as implemented in the SciPy library [49].

All NFS-60 cell proliferation assays were conducted as described above unless indicated otherwise. In live cell tracking assays, cells were monitored in the IncuCyte S3 Live-Cell Analysis System (Essen Bio) with a 10× objective. Cell proliferation was analyzed using IncuCyte S3 Software. For these experiments, NFS-60 cells were maintained at 5% CO2 and 37 °C in NFS-60-medium (RPMI 1640 medium with additional 2 mM L-glutamine, 1 mM Na-pyruvate, 10% FCS, 1% Pen-Strep, and 10 ng/ml rhG-CSF). Prior to the proliferation assay, the cells were washed 3 times with PBS and starved for at least 4 h in NFS-60 medium without rhG-CSF. The assay was then performed in an L-ornithine pretreated 96-well plate, with 45,000 cells seeded per well.

### NFS-60 competitive inhibition assay

The NFS-60 cells were cultured under the same condition as described for the NFS-60 cell proliferation assays with the following changes. After washing the cells 3 times with NFS-60-medium without KMG-2, a final constant concentration of 50 pg/ml of rhG-CSF (unless otherwise indicated in the figures) was applied to the cells together with varying concentrations of the designed proteins. The choice of rhG-CSF concentration mimics the reported physiological serum levels in healthy individuals [50]. Fluorescence measurements were acquired for each experimental condition and subsequently min-max normalized against control samples. These control samples consisted of cells treated with and without the constant concentration of rhG-CSF. From these values, the half-maximal inhibitory concentration (*IC*_*50*_) was determined in the same way as described for the *EC*_*50*_ parameter in the proliferation assays.

### Structure determination of bop1 using X-ray crystallography

Crystallization screens were set up with a Mosquito robot (TTP Labtech) in 96-well plates at 21 °C, using 50 μl of reservoir solution and sitting drops containing 400 nL of reservoir and 400 nL of protein solution of bop1 at a concentration of 9 mg/ml. Within 14 to 30 days, single crystals of bop1 grew in a condition with 0.1 M MES (pH 7.0), 20% (w/w) PEG 8,000. Single crystals were cryoprotected through the addition of 20% PEG 400, flash cooled and stored in liquid nitrogen until data collection. Diffraction data were collected at 100K on an EIGER 16M detector at beamline X10SA at the Swiss Light Source (SLS) at a wavelength of 1.000 Å. Data were processed and scaled using XDS [51] and the structures solved using molecular replacement with MOLREP [52] and the AF2 model of bop1 as a search model. The structure was completed by cyclic refinement with REFMAC5 [53] and modeling using Coot [54]. Data collection and refinement statistics, coordinates, and structure factors are deposited in the PDB under accession code 8QUP.

### Computationally guided affinity maturation

In order to further enhance the binding affinity of the primary binding site of bop1, we selected 11 amino acid positions for computational optimization. The structural models were built as a complex of each of the 17 NMR frames of Boskar4 (7NY0 [31]) structurally aligned to the G-CSF:G-CSFR complex (2D9Q [32]). Two starting template sequences of the ligand were based on the enhanced affinity variants bv6 and bv8 [34] and modeled separately into the complexes using Modeller [55], where each of the 2 variants was modeled into the 17 NMR frames, yielding a total of 34 modeled complexes. For each of these complexes, all 11 positions were mutated in silico to all proteinogenic amino acids (but G, P, and C) utilizing the combinatorial sampler of Damietta (v0.32; S1 Methods) [56]. The combinatorial design simulations were performed in 10 scrambled-order replicas for each modeled complex, where the swarm algorithm has effectively sampled a total of 2.3 × 10^9^ rotamers across all simulation replicas. This resulted into a total of 612 unique, low-energy designed sequences. The sequences of the starting variants bv6 and bv8 were added to this pool of unique sequences, resulting in a total of 614 sequences. The obtained amino acid sequences were split into 2 pools of contiguous fragments, the first encoding the binding protein till L55 (numbering of PDB:7NY0) and the second starting from D56 (number of PDB: 7NY0) till the end of the protein sequence. The 2 amino acid sequence pools were used to design two oligo pools encoding for the corresponding mutants. In total, oligo pool 1 covered 298 unique oligos, and oligo pool 2 included 330 unique oligos. These two pools of single-stranded DNA oligos were ordered (oPools Oligo Pools, IDT, Inc.) encoding the desired mutations on a forward and reverse primer pair (S1 Data) to generate a library with a maximum degeneracy and diversity of 9.8 × 10^4^. Finally, the library was generated by PCR using the 2 oligo pools to linearize an *E*. *coli* display system plasmid [57] encoding a fusion between the N-terminal part of intimin and Boskar4 (pNB4, Table B in S1 Text). The obtained PCR product was purified with the Wizard SV Gel and PCR Clean-Up System (A928, Promega) and blunt end ligation was performed followed by electroporation of freshly made competent *E*. *coli* DH10BT1R cells [58] to yield 6.0 × 10^6^ total transformants.

In more detail, prior to transformation in DH10BT1R cells, 0.4 U/μl T4 polynucleotide kinase was incubated with 5 ng/μl of linearize PCR product for 30 min at 37 °C, according to manufacturer instructions. After that, 0.25 U/μl T4 DNA ligase was added and incubated overnight at 16 °C. The ligation product was purified with the PCR Clean-Up System (A928, Promega) and eluted into 20 μl filtered ddH2O. The complete 20 μl were transformed into electrocompetent *E*. *coli* as described in Tu and colleagues [58]. Specifically, 50 ml of freshly grown *E*. *coli* (DH10B T1^R^) of an OD_600_ of ~0.6 was washed 2 times with 35 ml filtered ddH2O. After washing, the bacterial pellet was resuspended with the purified ligation mix. The electroporation was performed at 1,250 V and 5 ms. The cells were grown in 1 ml SOC Outgrowth Medium (B9020, NEB) for 2 h in a standard glass tube at 30 °C with 160 rpm. A 10-fold dilution series of the cells were made in SOC-medium and they were plated on agar plates containing 34 μg/ml chloramphenicol and 2% (w/v) D-glucose to estimate the total number of transformants. The rest of the sample was plated on 5 agar plates of the same type and incubated at 30 °C for about 20 h. The bacterial lawn was scraped from the plates and well mixed in 10 ml LB medium. The plasmids were isolated from 500 μl of bacterial suspension and the obtained plasmid library was analyzed by Sanger sequencing.

To perform cell sorting of the bacterial clones bound to G-CSFR, fluorescently labeled rhG-CSFR was prepared according to the protocol outlined in Salema and colleagues [57]. Specifically, a total of 100 μg of recombinant human G-CSFR (381-GR/CF, R&D Systems) was subjected to a reaction with Biotin-NHS (H1759, Sigma) in a 1:20 molar ratio (G-CSFR:Biotin-NHS), within a 950 μl solution of PBS. The mixture was incubated at RT for a duration of 2 h. Subsequently, the reaction was quenched by adding 50 μl of Tris (1 M pH 7.5). The resulting product underwent purification through a desalting column (Sephadex G25 PD-10, GE Healthcare), followed by concentration of elution fractions via ultrafiltration (3 kDa, UFC800324, Merck). The biotinylated rhG-CSFR (BioG-CSFR) was then aliquoted and stored at −20 °C until it was utilized for further experiments.

The screening process of the library also followed a modified protocol based on the procedure originally described in [57]. Initially, DH10BT1R *E*. *coli* cells were transformed using the display vector containing the protein library. The bacteria were grown on LB agar plates with 34 μg/ml chloramphenicol and 2% (w/v) D-glucose at 30 °C. After harvesting the plates, 1 ml of resuspended bacteria with an OD_600_ of 3.3 was washed twice with 1 ml LB and then diluted to a starting OD_600_ of 0.33 in 10 ml LB with 34 μg/ml chloramphenicol. The cells were grown at 30 °C and 160 rpm and after 1 h, 50 μm IPTG was added to the culture, and it was further grown for 3 h under the same conditions. Cells were collected (equal to 1 ml of a cell resuspension with an OD_600_ of 1), washed twice with 1 ml PBS and resuspended in 400 μl PBS. In the next step, 190 μl of this cell suspension was incubated with 10 nM BioG-CSFR at RT for 1 h, followed by a single wash with 1 ml PBS. The washed cell pellet was resuspended in 200 μl PBS, to which 0.375 μl PE-Streptavidin (405203, BioLegend) was added, and incubated at 4 °C for 30 min. Finally, the sample was washed once more with 1 ml PBS and resuspended in 1 ml PBS. The screening was performed using a BD FACSMelody Cell Sorter having set the PTM voltage for PE to 517 V, SSC to 426 V, and an SSC threshold of 673 V. No gating was applied, and 100,000 events were measured per sample. For sorting, 300 μl of the sample was mixed with 4 ml ice-cold PBS, and the flow rate was adjusted to achieve an event rate of approximately 8,000 events/sec. Approximately, the top 0.1% of the total population, based on an SSC-A/PE-A plot, was sorted into a 1.5 ml tube containing 200 μl LB medium, which was cooled to 5 °C. The sorting process continued until at least 20 times more events were screened than the complexity of the corresponding library. All sorted cells were plated on LB agar plates with 34 μg/ml chloramphenicol and 2% (w/v) D-glucose and were incubated at 30 °C for around 20 h. The harvested plates were then grown overnight in LB medium with 34 μg/ml chloramphenicol and 2% (w/v) D-glucose at 30 °C under static conditions. From this culture, 1 ml of resuspended bacteria with an OD_600_ of 3.3 was used for the next selection cycle performed in the same way and repeated for 5 iterative cycles. All flow cytometry results were analyzed using FlowJo v10.1 Software (BD Life Sciences).

Single colonies of bacteria pools which were enriched for 3 or 5 cycles of FACS (as described above) were grown overnight in 100 μl LB (supplemented with 34 μg/ml chloramphenicol and 50 μm IPTG) in a 96-well plate sealed with Breathe-Easy sealing membrane (Z380059, Sigma-Aldrich) at 30 °C with 1,100 rpm on a benchtop shaker (Thermomixer comfort, Eppendorf) covered with aluminum foil. At the next day, the cells were centrifugated at 3,200 g for 5 min, resuspended in 150 μl PBS, and centrifugated again. The washing was repeated one more time and the cells got resuspend in 50 μl PBS containing 10 nM BioGCSFR. After the plate was incubated for 1 h at RT, the cells were washed one time with 200 μl PBS and resuspended in 50 μl PBS, and 25 μl of the resuspended cells were mixed with 25 μl PBS containing 1.25 μg/ml Avidin-HRP conjugate (Invitrogen, 434423) followed by a 30-min incubation at 4 °C in the dark. Afterwards, the cells were washed one time with 200 μl PBS and resuspended in 100 μl PBS. The cell density (OD_600_) of each well was measured with the plate reader. In a separate dark plate (655097, Greiner Bio One), 12.5 μl cell suspension was added to 75 μl PBS. Then, 12.5 μl ECL-substrate-solution (1705061, Bio-Rad) was pipetted quickly into each well; the plate was sealed with a parafilm and mixed vigorously for 10 s on a vortex orbital shaker. Then, the luminescence of each well was measured on the plate reader. The luminescence signal of each variant in each well was analyzed by normalizing to the corresponding cell density and calculating a Z-score for each sample over all wells of the same condition. From the top 5 variants of each condition, plasmids were isolated with a plasmid preparation kit (740588, Macherey-Nagel) from a 3 ml overnight culture grown at 30 °C with 160 rpm in LB with 34 μg/ml chloramphenicol and 2% (w/v) D-glucose. Afterwards, the acquired plasmids were sequenced, and distinct Boskar4 variant (bv) genes were identified which were designated as bv21, bv22, bv23, and bv24. The Alignment consensus logos of the mutated sites were illustrated using AlignmentViewer [59]. For protein purification and following analysis, single Boskar4 variant genes were amplified by PCR from the pN vector [57] and ligated between NdeI and XhoI replacing Boskar4 of pET28a.

### Competitive inhibition assays in Kasumi-1 AML cell line

Kasumi-1 cells [60] were cultured at 5% CO_2_ and 37 °C in RPMI 1640 medium supplemented with 1 mM L-glutamine, 20% FCS, and 1% Antibiotic-Antimycotic (15240062, Gibco). In a black 96-well plate, 40,000 cells were seeded in each well with a constant concentration of 400 pg/ml of rhG-CSF, alongside varying concentrations of the designed proteins. The cells were then incubated under standard conditions for 72 h. CellTiter-Blue Reagent was added into each well, and the resulting fluorescence was measured as described in the NFS-60 cell proliferation assay. The fluorescence values obtained were subsequently normalized to the cells treated with 400 pg/ml of rhG-CSF. Finally, the *IC*_*50*_ was determined as described above. Leave-one-out fitting was used to calculate the mean and standard deviation of the *IC*_*50*_ across the 3 biological replicates. This approach was chosen because the biological replicates encompassed varying numbers of treatments.

### Evaluation of dose-dependent effects of inhibitors on the proliferation of CD34^+^ HSPCs

Human, rhG-CSF-mobilized CD34^+^ cells were obtained from cryopreserved stem cell bags. After thawing, CD34^+^ cells were cultured for up to 5 days in Stemline II Hematopoietic Stem Cell Expansion medium (Sigma Aldrich; #50192) supplemented with 10% FBS, 1% penicillin/streptomycin, 1% L-glutamine and 20 ng/ml IL-3, 20 ng/ml IL-6, 20 ng/ml TPO, 50 ng/ml SCF, and 50 ng/ml FLT-3L. Before experimentation, cells were washed twice in ice-cold PBS and 2 × 10^4^ cells/well were incubated in poly-L-lysine–coated 96-well plates for 5 days in an IncuCyte S3 Live-Cell Analysis System (Essen Bio) with a 10× objective at 37 °C and 5% CO_2_. Cells were incubated in the same Stemline II Hematopoietic Stem Cell Expansion medium supplemented with 10% FBS, 1% penicillin/streptomycin, 1% L-glutamine, and 1 ng/ml of rhG-CSF or PBS in the presence or absence of designed inhibitors (i.e., bop1 or bop3) or an inert helical protein (moevan_control [61]), at concentrations ranging from 5 μg/ml to 0.1 μg/ml (as indicated in the respective figure). Cell proliferation was analyzed using IncuCyte S3 Software.

## Results

### Design of single-domain, bifaceted G-CSFR binders

In this study, we used Boskar4 as our starting design template to build G-CSFR inhibitors. Boskar4 is a de novo designed protein domain that is small (13 kDa), proteolytically and thermally stable, and binds to G-CSFR at the same binding site of the native ligand, G-CSF [31]. Previously, we showed potent G-CSFR agonists can be made by tandemly repeated Boskar4 fusions, which dimerize and activate the receptor [31]. Through this work, we pursue the inverse goal of creating G-CSFR inhibitors based on the single-domain Boskar4 template. Based on our previous work to create rigid Boskar4-based dimerizers that can associate G-CSFR in non-native geometries, we observed that the inter-transmembrane domain (inter-TMD) spacing of the receptor subunits affects its activity [34]. Specifically, ligands that impose 2:1 receptor:ligand complexes with longer inter-TMD spacing not only result in weaker activity, but also substantially reduced receptor dimerization levels [34]. We ascribe the latter to severely compromised recruitment of the second receptor subunit at the cell surface caused by the unfavorable orientation of the second binding site. We therefore expect that designs further distorting the native receptor orientation would preferably be recruited in a 1:1 stoichiometry. An additional challenge to this end relates to identifying Boskar4 variants that are exclusively monomeric in solution to avoid any unintended oligomerization, and thus unintended activation, of the receptor. Therefore, here we sought to modify the Boskar4 domain towards the 2 pronged-goal of reducing its ability to associate the receptor in a favorable orientation, and reducing its self-interactions by repurposing more of the ligand’s surface area for receptor binding. Given these objectives, in addition to the existing (i.e., primary) receptor-binding site, we sought to graft a secondary binding site on the Boskar4 surface. AlphaFold3 predictions indicated juxtaposing the secondary binding site yields inter-TMD spacings of 214 ± 18 Å and 251 ± 9 Å for parallel and antiparallel orientations, respectively. This spacing is substantially larger than that predicted for the full-length G-CSF:G-CSFR complex 84 ± 43 Å and could lead to strained ternary complexes in membrane embedded receptors (Figs A and B in S1 Text).

In a previous study, we reported an enhanced variant (5-point mutant) of Boskar4, termed bv6, which binds 12-fold tighter to G-CSFR, and functions as a more potent granulopoietic agonist when tandemly repeated in a two-domain fusion [34]. Given its already superior properties to Boskar4, we based our subsequent protein engineering on this design. Whereas the primary binding site lies on H2 and H3 (i.e., second and third helix of bv6), the secondary binding site would reside on H1 and H4 (i.e., first and fourth helix of bv6) in either parallel or antiparallel orientations to the primary site. We initially created 4 graft variants with small (12 residues) or large (15 or 17 residues) patches as the secondary binding sites. The resulting AlphaFold2 models had predicted backbone root mean square deviations (RMSDs) from the corresponding binding site of G-CSF (PDB: 2D9Q) to range between 0.6 and 0.7 Å and between 1.2 and 1.5 Å, for the primary and secondary binding sites, respectively (Figs C and D in S1 Text). We refer to these 4 designs as *boa* or *bop* (bivalent-orientation antiparallel or parallel), where boa/p1 or boa/p2 refer to the designs with either the small or the large secondary binding patches, respectively (Tables A and B in S1 Text).

### The bifaceted designs are hyper-stable and monomeric in solution

**Fig 1:**
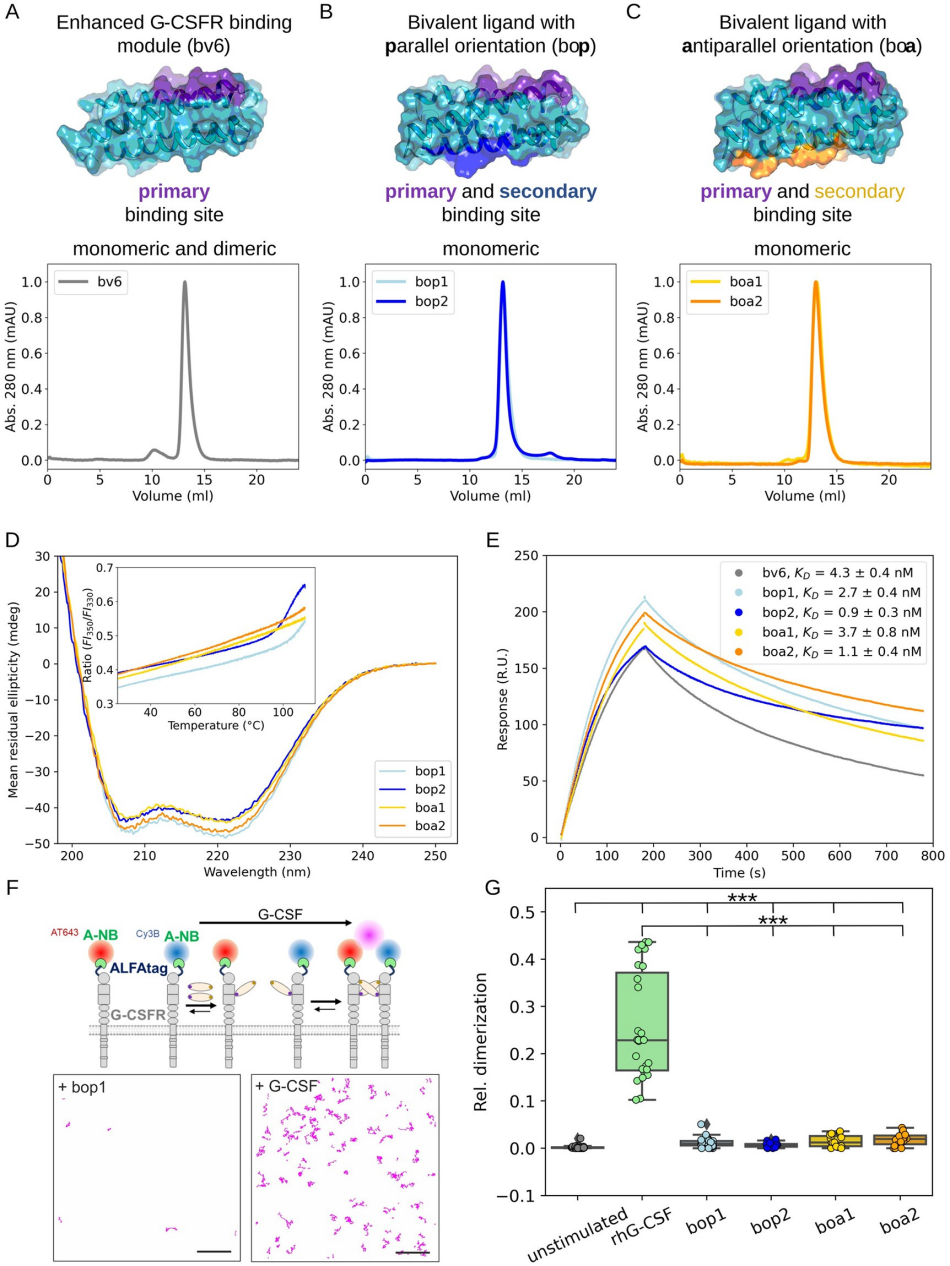
The bifaceted designs exhibit stronger binding affinity yet reduced receptor-dimerization capacity. **(A)** Analytical SEC shows the design template bv6 (an enhanced-affinity Boskar4 variant [34]) to be minorly dimeric in solution, in contrast to the bifaceted designs which appear exclusively monomeric in solution **(B, C)**. Prediction models of the ternary complexes assembled by the bifaceted designs are shown in Fig A in S1 Text. **(D)** All the designs have strong helical CD spectra and are highly thermostable (inset; nanoDSF melting curves), where the bop designs are less stable (i.e., melting onset at 100 °C) in comparison to the boa designs. NanoDSF plots show the mean of ratio of the fluorescence intensity at 350 nm and 330 nm from 4 technical replicates (Fig F in S1 Text). **(E)** Analysis of SPR sensograms (Fig G in S1 Text) indicates that all the bifaceted designs bind rhG-CSFR with higher affinity than the starting template, bv6. Particularly, designs with larger secondary binding interfaces (bop2 and boa2) bound 4-fold tighter than bv6. The binding affinity (*K*_*D*_) values were obtained from fitting 2-fold dilution series performed under the same conditions (Fig G and Table C in S1 Text). **(F)** G-CSFR dimerization was quantified in live HeLa cells by dual-color single molecule imaging of ALFA-tagged G-CSFR labeled through anti-ALFA nanobodies conjugated with 2 different dyes (top) and co-tracking analysis (bottom). **(G)** Comparison of the relative number of co-tracked G-CSFR observed for untreated cells and after addition of agonist or antagonists (10 nM each). Each data point represents the result from 1 cell. *P*-values were obtained from a two-sample Kolmogorov–Smirnov test (*** indicates *p* ≤ 0.001). Numerical data for graphical items in Fig 1D, 1E, and 1G can be found in S1 Data. CD, circular dichroism; G-CSFR, granulocyte-colony stimulating factor receptor; nanoDSF, nanoscale-differential scanning fluorimetry; SEC, size exclusion chromatography.

The expression of all 4 designs in *E*. *coli* resulted in a high soluble protein yield, which was readily purified to homogeneity using IMAC and preparative SEC. Analytical SEC showed the purified proteins (bop1/2 and boa1/2) to be strictly monomeric in solution (Fig 1A–1C). This soluble, monomeric property is advantageous for obtaining inhibitors that do not inadvertently aggregate or oligomerize G-CSFR. This is in contrast to the single-domain form of the original design, Boskar4, which partitions between monomer and dimer in solution (Fig E*B* in S1 Text), and even its isolated monomeric fraction could only weakly inhibit G-CSFR activation (Figs E*C* and E*D* in S1 Text). The starting template in this study, bv6, also showed a tendency to form dimers in solution, similar to Boskar4 (Fig 1A), which explains its residual capacity to activate G-CSFR (Fig E*E* in S1 Text).

CD measurements revealed strong α-helical profiles for all designs indicating their well-folded nature (Fig 1D). Additionally, nanoDSF showed all designs to be hyper-thermostable. During the temperature ramp, none of the designs showed increased scattering up to 110 °C, highlighting the colloidal stability of the proteins (Fig F in S1 Text). For boa1 and boa2, the fluorescence ratio (350 and 330 nm) was unchanged during the heating ramp, while for bop1 and bop2 it indicated a melting onset beyond 90 °C. However, the cooling ramp of bop1 and bop2 (from 110 °C to 25 °C) showed this melting signal onset to be reversible.

### The designs strongly bind and minimally dimerize G-CSFR

We then set out to evaluate the binding affinities of the bifaceted designs to G-CSFR using surface plasmon resonance, which was done in a direct comparison to their starting template bv6. The results revealed a pattern whereby all the bivalent designs were more affine than the starting template, and the large-patch grafts (boa2 and bop2) bound G-CSFR tighter in comparison to the small-patch grafts (boa1 and bop1). Specifically, bv6 showed the weakest binding affinity (*K*_*D*_ = 4.3 ± 0.4 nM) in comparison to the other designs (Fig 1E, and Fig G and Table C in S1 Text), which points to the putative role of the secondary binding site in reducing the apparent dissociation rate (*k*_*d*_). Regardless of the orientation of the secondary binding site, its small-patch variants, boa1 (*K*_*D*_ = 3.7 ± 0.8 nM) and bop1 (*K*_*D*_ = 2.7 ± 0.4 nM), displayed weaker receptor-binding affinity than their large-patch counterparts; boa2 (*K*_*D*_ = 1.1 ± 0.4 nM) and bop2 (*K*_*D*_ = 0.9 ± 0.3 nM). These results highlight the symmetric regularity of the template helical bundle, whereby the orientation of the secondary binding site was less important than the graft size in improving the interaction with the receptor.

To explore residual capacity of the designs to dimerize G-CSFR in the plasma membrane, we applied live-cell single-molecule imaging. For this purpose, we expressed G-CSFR with an N-terminal ALFA-tag in HeLa cells for cell surface selective labeling with anti-ALFA nanobodies conjugated to 2 different fluorescent dyes (Fig 1F). Upon dual-color single-molecule imaging by TIRF microscopy, G-CSFR dimerization was monitored and quantified by co-tracking analysis, using cells with comparable receptor cell surface densities (Fig 1F and Fig H*B* in S1 Text). Strikingly, all the bifaceted designs induced significantly reduced receptor dimerization in comparison to rhG-CSF. Nonetheless, it was significantly increased compared to untreated cells, but did not exceed 5% receptor dimerization, indicating a minor capacity of all designs to induce residual receptor dimerization (Fig 1G). In line with these observations, the diffusion coefficient of G-CSFR was only significantly reduced upon treatment with rhG-CSF, but not for the antagonistic designs (Fig H*A* in S1 Text).

### The designs inhibit G-CSFR activation by different magnitudes

**Fig 2:**
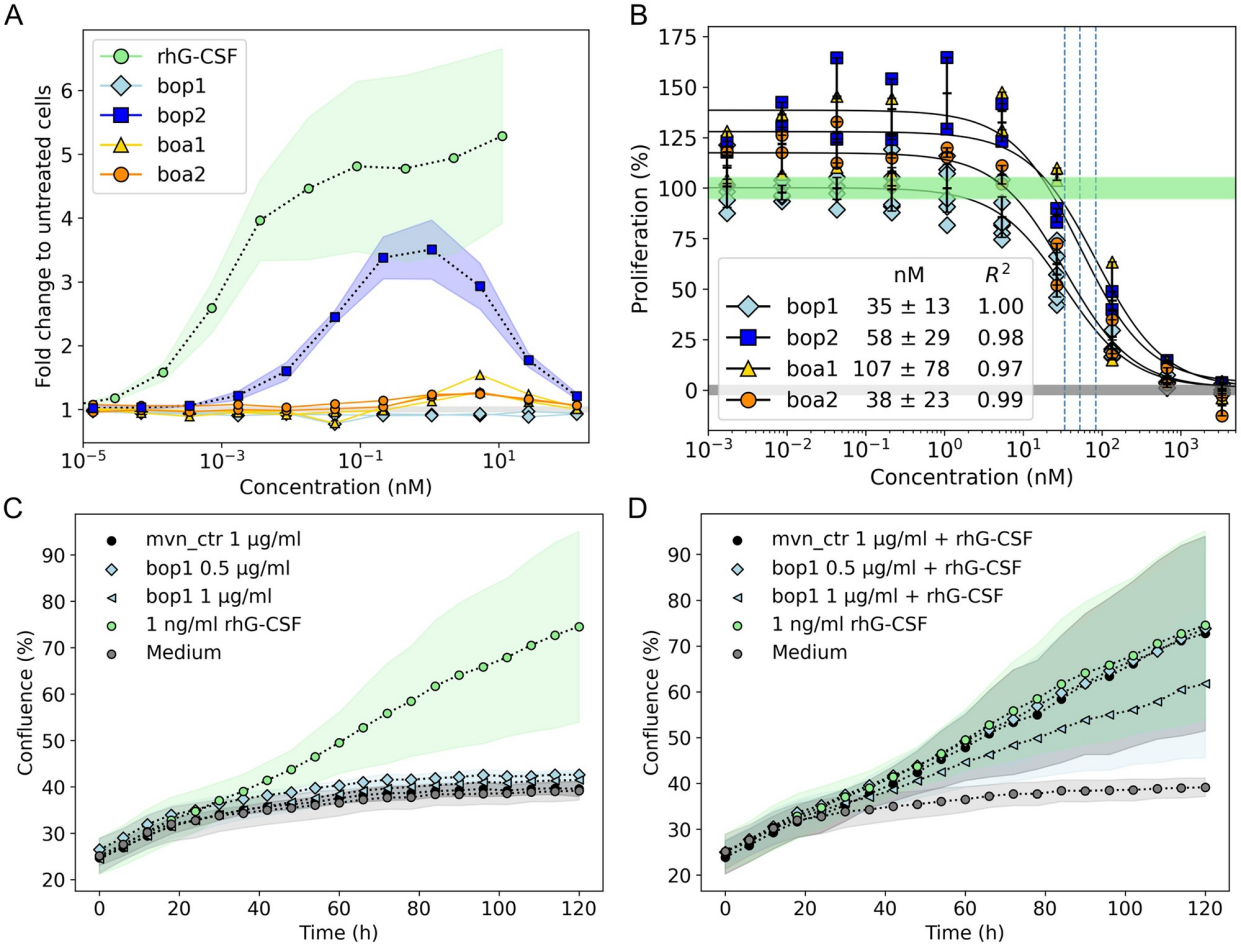
Proliferation assays in a reporter cell line and primary cells identify bop1 as the strongest inhibitor of G-CSF activity. **(A)** The proliferative activity (fold-change to untreated cells) of the designs in NFS-60 cells is lower in comparison to rhG-CSF (data for cell activity of rhG-CSF was taken from [34]). Specifically, bop2 has the highest activity, whereas boa1 and boa2 have low residual activity, and bop1 has no detectable residual activity across the entire concentration range. The proliferation results were obtained from 3 (lines: mean, shades, standard deviations) or 2 (individual line plots) independent biological replicates. **(B)** Competitive inhibition of rhG-CSF-induced proliferation indicates bop1 to be the most potent in inhibiting G-CSF activity in NFS-60 cells. Unlike other designs, bop1 had no residual activity at sub-inhibitory concentrations. A constant concentration of rhG-CSF (50 pg/ml; 100% proliferation) was used in all conditions, where the green (100%) and gray (0%) shades cover the mean and standard deviation of normalized cell proliferation with and without rhG-CSF, respectively. At least 2 biological replicas were performed for each design, where half-maximal inhibitory concentrations (*IC*_*50*_) values were obtained from the corresponding fit. **(C, D)** The bop1 design does not proliferate HSCPs, but inhibits the proliferative effect of rhG-CSF. Proliferation assays using 0.5 μg/ml (36 nM) and 1 μg/ml (72 nM) of bop1 were performed on healthy donors’ CD34+ HSPCs either without **(C)** or with the addition of 1 ng/mL rhG-CSF **(D)**. As a negative control, 1 μg/ml of Moevan_control (mvn_ctrl, [61]) was tested, which is a protein lacking a functional G-CSFR binding site. Shown is mean and standard deviation of 2 independent replicates of 3 technical replicates each. Numerical data for graphical items in Fig 2A–D can be found in S1 Data. G-CSFR, granulocyte-colony stimulating factor receptor; HSPC, hematopoietic stem and progenitor cell.

To establish a baseline on G-CSFR activation potential by the designs, we first tested their proliferative activity in a G-CSF-responsive cell line (NFS-60). These experiments showed partial activation by 1 design, which displayed a parallel, large secondary binding site (i.e., bop2), which was bell-shaped due to self-competition at higher concentrations and exhibited lower *EC*_*50*_ and *E*_*max*_ than rhG-CSF (Fig 2A and Fig I in S1 Text). Two other designs (boa1 and boa2) showed only a marginal increase in proliferation within a narrow concentration window between 5 and 10 nM. We sought to assess the impact of parallel versus antiparallel orientations on receptor activation by creating short tandem two-copy (st2) constructs of bop2 and boa2 (Fig J in S1 Text). NFS-60 cell proliferation experiments demonstrated that bop2_st2 agonists are significantly more active compared to boa2_st2 agonists (Fig J*D* and Table C in S1 Text). Interestingly however, one design (bop1) showed no measurable residual activity (Fig 2A). To evaluate the inhibitory potential of our designs, we followed up by conducting competitive inhibition assays of rhG-CSF in NFS-60 cells (Materials and methods). At concentrations above 10 nM, all designs showed substantial inhibition of rhG-CSF-induced proliferation, with average *IC*_*50*_ values ranging from 35 to 107 nM (Fig K and Table C in S1 Text). However, at lower concentrations, bop2, boa1, and boa2 showed different levels of design-induced residual proliferation, which was not the case for bop1 (Fig 2B, and Fig K*B* in S1 Text). Indeed, bop1 treatment resulted in no residual activation at low concentrations, and possessed the highest inhibitory potency (*IC*_*50*_ = 35.3 ± 12.9 nM; *n* = 6). To validate the specificity of this inhibitory activity, we evaluated the ability of bop1 to outcompete higher concentrations of rhG-CSF in a concentration-dependent manner, which was true for above-physiological concentrations (100 and 200 pg/ml) of G-CSF [50] (Fig L in S1 Text). Moreover, we conducted proliferation assays in primary human CD34^+^ hematopoietic stem and progenitor cells (HSPCs). As expected, there was no observable proliferative activity when the cells were treated with up to 1 μg/ml bop1 (Fig 2C). Conversely, an inhibition of HSPCs stimulated with 1 ng/ml rhG-CSF and treated with 1 μg/ml bop1 was evident (Fig 2D). Therefore, we selected bop1 as the design candidate to further analyze it and enhance its inhibitory potency.

### The structure of bop1 matches the design model at atomic accuracy

**Fig 3:**
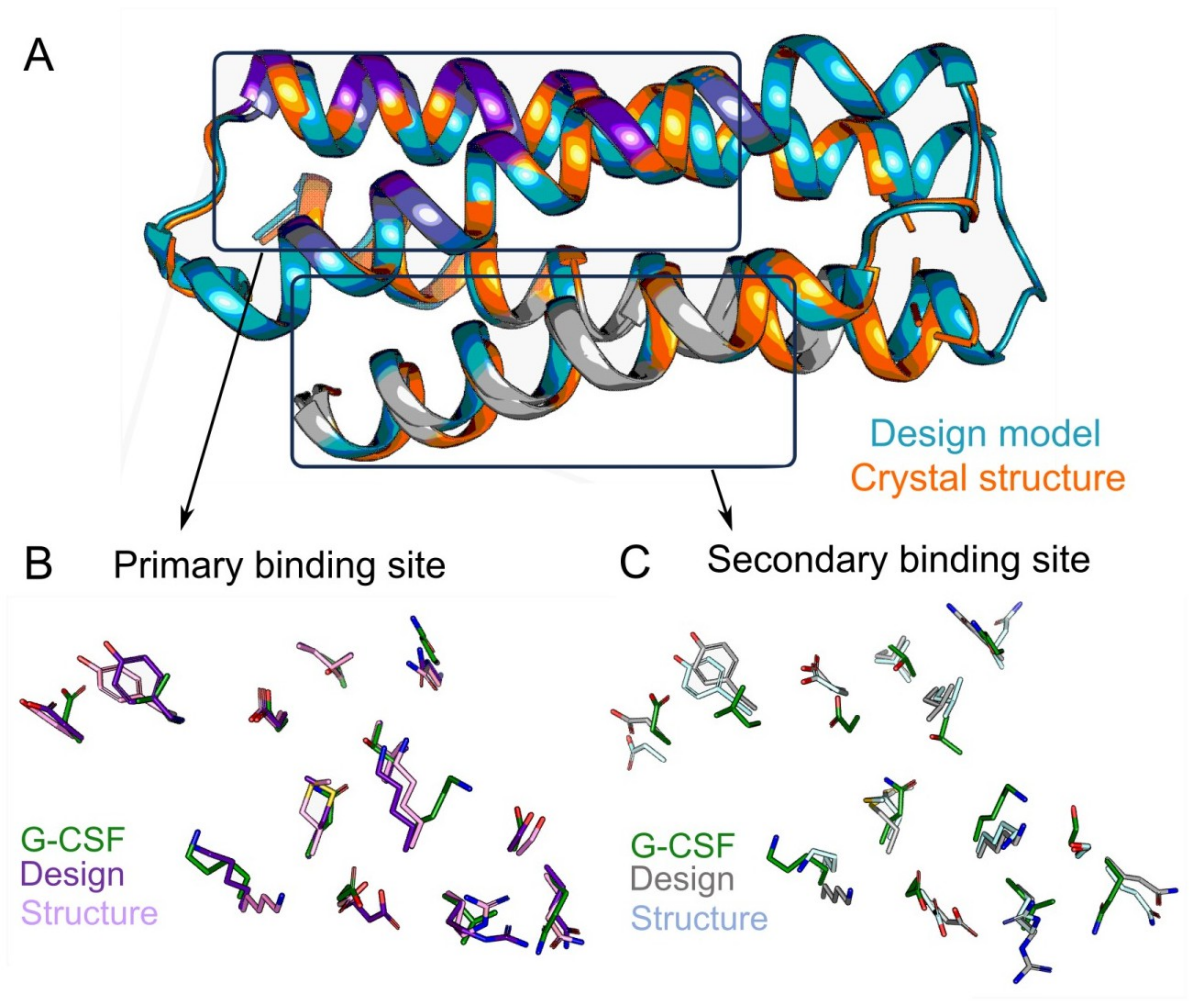
Crystal structure of bop1 matches its design model at atomic resolution. **(A**) Alignment of the crystal structure of bop1 (orange) with the design model (teal) shows a global backbone RMSD of 1.03 Å. Primary and secondary binding site helices are colored in purple and gray, respectively. **(B, C)** Backbone RMSD of the binding sites show the primary binding site of the design model and the crystal structure to align better to the corresponding positions in the G-CSF structure (0.6 Å; PDB: 2D9Q) than the secondary binding site (1.2 Å). RMSD, root mean square deviation.

To judge the design accuracy, we sought to solve the crystal structure of bop1. We obtained single crystals diffracting to 1.3 Å, with one copy of bop1 in the asymmetric unit, which could be solved by molecular replacement using an AlphaFold2-generated design model (Table D in S1 Text). Overall, the crystal structure matched the design model to atomic accuracy, with a global backbone C^α^ RMSD between the structure and the model of 1.03 Å (Fig 3A). The comparison between the primary binding site residues of bop1 and the native receptor-binding site II of G-CSF (PDB: 2D9Q) showed only minor backbone C^α^ RMSD of 0.6 Å (Fig 3B). The alignment was worse however when comparing the secondary binding site residues of bop1 to site II of G-CSF, which yielded and RMSD of 1.2 Å (Fig 3C). While these results emphasize the weaker structural match, as was expected from the design model, the crystal structure showed no unexpected conformational deformities. The inability of bop1 to dimerize G-CSFR in cells might thus be attributed to the combined effect of its monomeric nature, weak receptor interactions by the secondary binding site, and the predicted far inter-TMD spacing when compared to G-CSF.

Taken together with the excellent agreement of the bop1 design model and its experimental structure, as well as its superior stability, solubility, and monomeric nature, we were motivated to further improve its inhibitory potency.

### Computationally guided enhancement of bop1 inhibitory potency

**Fig 4:**
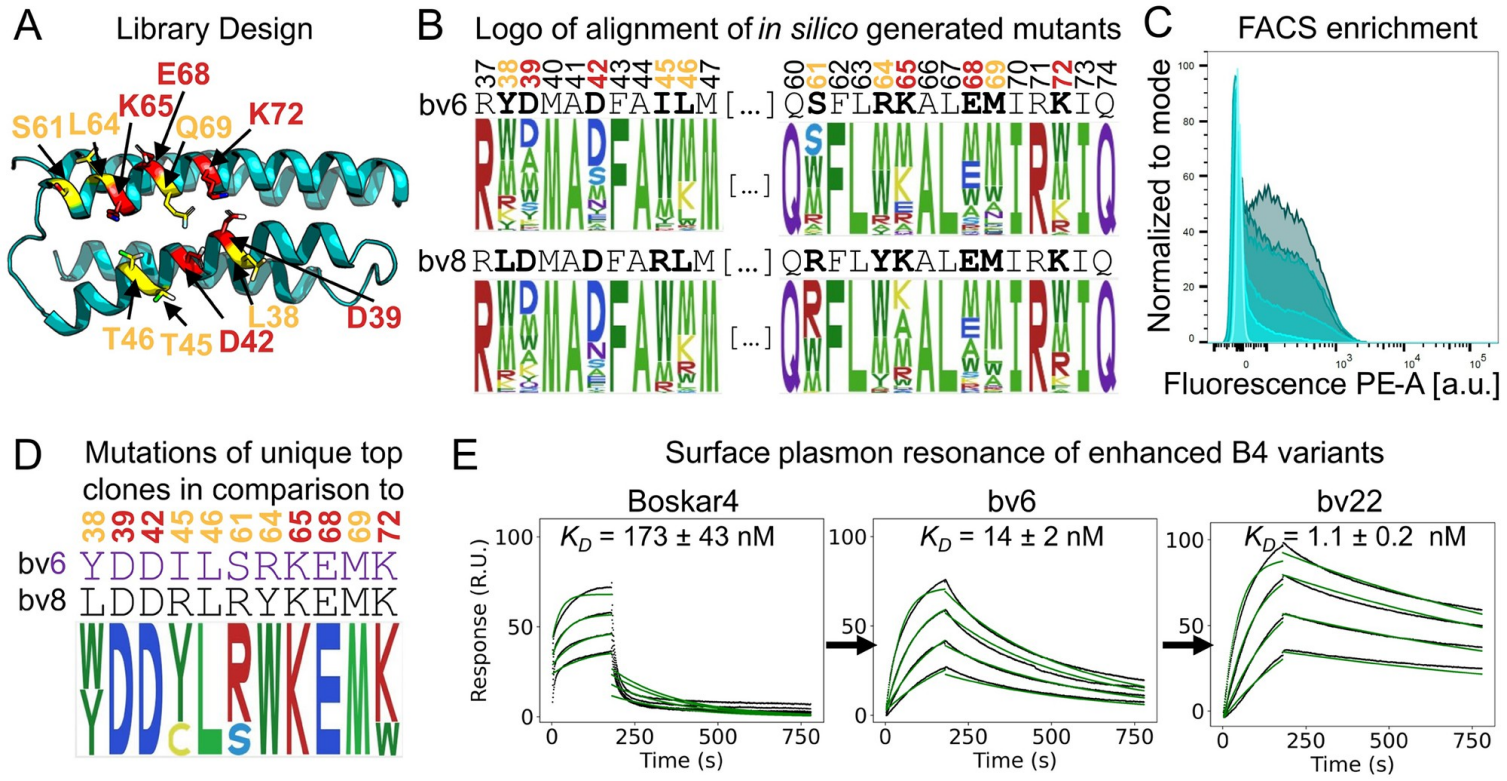
Computationally guided affinity enhancement of bop1. **(A)** Position on the Boskar4 structure subjected to optimization in the modeled complex with G-CSFR. In total, 11 residues were optimized (orange or red). These included 5 positions identified to be critical for G-CSFR binding based on an alanine-scanning study of G-CSF (red, [62]). **(B)** Comparison between the mutants generated by the Damietta combinatorial sampler [56] in a logo that contains the alignment of the mutated sites. **(C)** High-throughput screening of the bacterial display library resulted in a strong FACS-enrichment of better binding clones. **(D)** Sequence logo of the top 10 selected clones. **(E)** SPR measurements of G-CSFR binding to Boskar4 (Original design), bv6 (first-generation variant), and bv22 (second-generation variant) demonstrate the enhancement in binding affinity over the first and second round of computationally guided affinity maturation (Fig N and Table C in S1 Text). Numerical data for graphical items in Fig 4C and 4E can be found in S1 Data. FACS, fluorescence-activated cell sorting; G-CSFR, granulocyte-colony stimulating factor receptor.

To improve the inhibitory potency of bop1, we reasoned that enhancing the affinity of only the primary binding site of bop1 can result in stronger G-CSF out-competition, without altering the monomeric nature of bop1, and thus its inability to substantially dimerize G-CSFR. We performed combinatorial design, setting 11 selected amino acid positions on surface of Boskar4 variants (bv6 and bv8) as mutable and 24 positions as repackable. These positions lie across the modeled Boskar4:G-CSFR binding interface and were designed using the Damietta software [56] (Fig 4A and S1 Methods). We then created a library from the sequence profile of lowest energy designs (Fig 4B and Fig M*A*-*B* in S1 Text; Materials and methods). The resulting library of binders was screened using a bacterial display system (Fig 4C and Fig M*C* in S1 Text; Materials and methods). Five sequential cycles of fluorescence-activated cell sorting (FACS) enriched the bacterial clones displaying binders to fluorescently labeled rhG-CSFR. After the third and fifth sorts, 48 single clones per condition were screened on plate-based assays to identify the clones with the tightest relative binding signal (Fig M*D* in S1 Text). From the 96 analyzed clones, the top 10 clones were sequenced. We identified 4 unique enhanced Boskar4 variants, named bv21, bv22, bv23, and bv24. Within these variants, we observed that 7 mutated positions have reverted to the starting sequence, indicating the near-optimal sequence of the primary binding site of the bv6 and bv8 variants (Fig 4D).

The new unique variants bv21, bv22, bv23, and bv24 encoding only the primary binding site (Table B in S1 Text) were expressed, purified, and biophysically characterized. All these variants were readily produced, well-folded, and highly thermostable (Fig N in S1 Text). Receptor-binding assays performed to compare the binding affinity of Boskar4, bv6, bv8, and the new variants (i.e., bv21-24), showed all the new variants to possess higher affinity. Specifically, all the new variants possessed a dissociation constant (*K*_*D*_) within the range of 1 nM to 2 nM, compared with 14 ± 2 nM for bv6, 26 ± 3 nM for bv8, and 173 ± 43 nM for the original Boskar4. Notably, bv22 demonstrated the highest affinity among the variants with a *K*_*D*_ of 1.1 ± 0.2 nM (Fig 4E and Fig O*A-G* and Table C in S1 Text). In their existing form (i.e., encoding only the primary binding site), the new variants already exhibited stronger G-CSFR inhibition in competitive inhibition assays. For example, bv22 had an *IC*_*50*_ of 35 nM, compared to 93 nM for bv6, and 2.3 μm for Boskar4. As observed previously, all tested designs, except for bop1, displayed residual activity at non-inhibiting concentrations (Fig O*H* in S1 Text).

**Fig 5:**
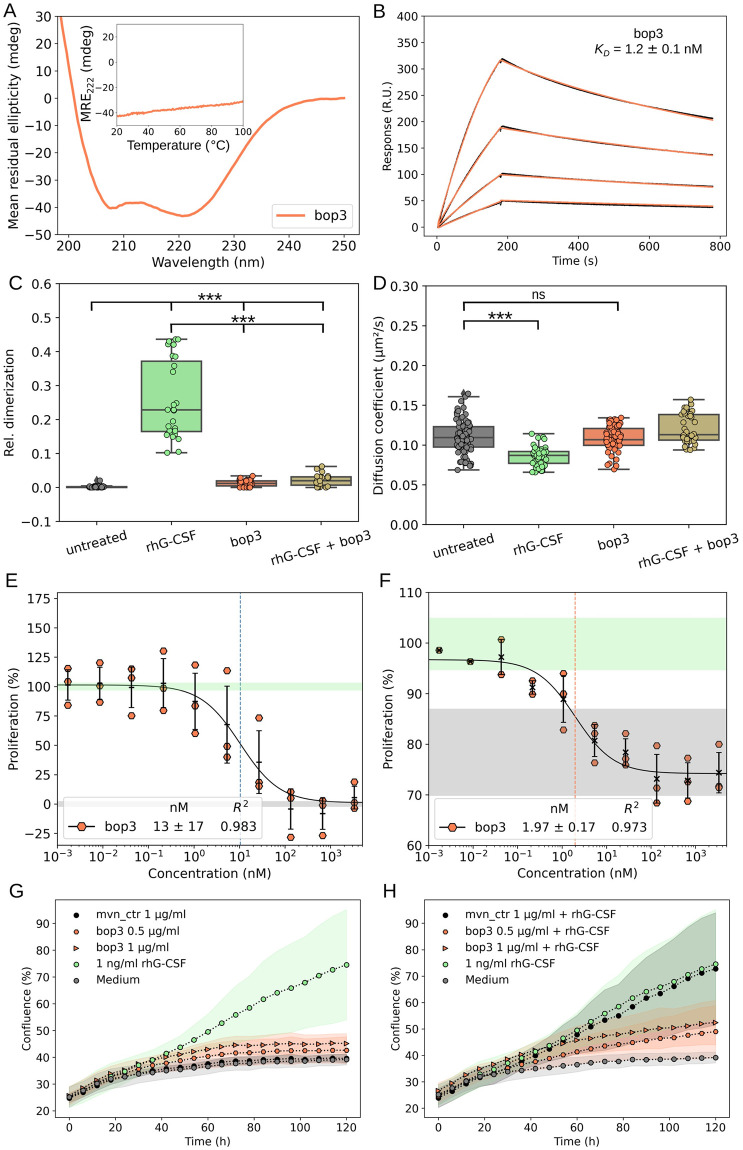
Characterization of the functionally enhanced inhibitor bop3. **(A)** Circular dichroism spectrum and melting curve confirm the helical and hyper-thermostable properties of bop3. Inset shows mean residual ellipticity at 222 nm between 20 and 100 °C, with no unfolding transitions, which is supported by similar nanoDSF (Fig O*B*). **(B)** SPR measurements also confirm a 10-fold improvement in the receptor-binding affinity. The pane shows sensograms from a 2-fold dilution series starting from 20 nM of bop3 against chip-immobilized G-CSFR. **(C, D)** Single-molecule imaging of G-CSFR dimerization in live cells: dimerization of G-CSFR upon stimulation with rhG-CSF, bop3 and a mixture of bop3 (100 nM) and rhG-CSF (10 nM) as quantified by co-tracking analysis (**C**) and corresponding diffusion constants **(D)**. Results from untreated cells are shown as a negative control. Each data point represents the result from one cell. *P*-values were obtained from a 2 Sample Kolmogorov–Smirnov test (***: *p* ≤ 0.001, ns: *p* > 0.05). **(E)** Competitive inhibition assays in NFS-60 cells show bop3 to be approximately 3-fold more potent in inhibiting G-CSF–induced proliferation than bop1, with no observed residual activity (Fig O*D*). **(F)** Competitive inhibition of G-CSF-induced proliferation in the Kasumi-1 AML cell line using bop3. In both NFS-60 (E) Kasumi-1 cells (F) 3 independent experiments were performed, and the half-maximal inhibitory concentrations (*IC*_*50*_ in nM) were obtained from the corresponding fit. In both assays, a constant concentration of rhG-CSF was employed, with 50 pg/ml in (E) and 400 pg/ml in (F), each corresponding to 100% proliferation. **(G, H)** CD34+ HSPCs proliferation assays using 0.5 μg/ml or 1 μg/ml of bop3 either without (G) or with the addition of 1 ng/ml rhG-CSF (H). Moevan_control (mvn_ctrl) was used as negative control. Shown is mean and standard deviation of 2 independent replicates, with 3 technical replicates per each independent replicate. Numerical data for graphical items in Fig 5A–H can be found in S1 Data. G-CSFR, granulocyte-colony stimulating factor receptor; HSPC, hematopoietic stem and progenitor cell; nanoDSF, nanoscale-differential scanning fluorimetry.

Finally, in order to combine the strong binding of bv22 and the eliminated residual activation of bop1 we created the bop3 design, which encodes the primary binding site of bv22 and the secondary binding site of bop1. The bop3 design was readily produced and purified, well-folded, and hyper-thermostable (Fig 5A and Fig P*A-B* in S1 Text). SPR titrations of bop3 showed it to bind G-CSFR with a *K*_*D*_ of 1.2 ± 0.1 nM (Fig 5B), which is comparable to that observed for bv22 (*K*_*D*_ = 1.3 ± 0.1 nM; Fig P*C* in S1 Text). Single-molecule co-tracking experiments also confirmed that bop3 effectively outcompetes rhG-CSF (10 nM) at 10-fold higher concentration (100 nM bop3) on the plasma membrane, abolishing the rhG-CSF–induced receptor dimerization (Fig 5C and 5D). In line with this, the proliferation assays in NFS-60 cells indicated no proliferative activity for bop3 across a broad range of concentrations (Fig P*D* in S1 Text). Competitive inhibition assays in the same cells showed bop3 to exhibit 3-fold improvement in inhibitory potency compared to bop1 (*IC*_*50*_ = 13.4 ± 16.9 nM; Fig 5E and Fig P*E* in S1 Text). We hence further evaluated the potential of bop3 to inhibit a G-CSF–dependent acute myeloid leukemia (AML) cell line, Kasumi-1 [60]. The results of this competitive inhibition assay showed bop3 exhibits an *IC*_*50*_ of 1.97 ± 0.17 nM (Fig 5F). Finally, we sought to repeat both the proliferation and competitive inhibition assays in the healthy donor-derived CD34^+^ cells. As expected, no discernible proliferative activity was observed following treatment with bop3 alone at concentrations of 0.5 μg/ml or 1 μg/ml (Fig 5G). In contrast, a pronounced inhibition of HSPCs proliferation stimulated with 1 ng/ml rhG-CSF was apparent at the same concentrations of bop3 (Fig 5H). A moderate inhibitory effect was noticeable even at the lower concentration of 100 ng/ml of bop3 (Fig P*F* in S1 Text). An inert protein with a similar structure (moevan_control [61]) was also purified and tested under the same conditions and did not show any measurable activity at the highest used concentration (i.e., 1 μg/ml; Fig 5G and 5H). Furthermore, bop3 was not toxic to any of the used cells, highlighting the specificity of its G-CSFR antagonism.

## Discussion

Inhibiting cytokine signaling is more safely achieved through a receptor antagonist rather than by blocking its soluble cytokine ligand. As receptor antagonists can be retrofitted with homing fusions to achieve local targeting effects, they can thus avoid systemic inhibition of cytokines, which are multirole regulators [63]. However, the challenge of blocking homodimeric cytokine receptors is two-fold. The first relates to the inhibitor’s capacity to outcompete the binding avidity of the bivalent ligand [64]. This can be further complicated by oncogenic mutations enhancing the dimerizing interactions between the receptor subunits [65] or its associated JAKs [38]. The other challenge pertains to avoiding the formation of dimeric (or higher oligomeric) states of the antagonist in solution, which can in turn form assemblies with signaling-compatible geometries. Such inadvertent signaling can yield a mixed biological effect (i.e., activation and inhibition) in vitro or in vivo. Therefore, the tendency of, for example, antibodies and the parent cytokine (here G-CSF) to self-associate [66–69] precludes them from providing suitable templates for inhibitor design.

In this study, we aimed to overcome these challenges by designing a bivalent ligand with extremely distorted receptor-binding geometry. This was achieved by starting from a de novo G-CSFR-binding domain (Boskar4) as a template. Boskar4 was designed to encode a single receptor-binding site, but was found to partition between monomeric and dimeric forms in solution [31]. Hence, our first step was to graft a secondary G-CSFR-binding site onto the same domain in 2 different orientations, with the three-pronged aim of: (i) enhancing its effective binding affinity to the receptor (through increased reassociation likelihood); (ii) minimizing the receptor activation probability by imposing an unfavorable (strained) orientation for dimerized receptor subunits; and (iii) disfavoring ligand dimerization in solution (at least in the presence of the receptor) by encoding receptor-binding information on a larger fraction of its solvent exposed surface area. All the bifaceted designs had stronger binding affinity compared with the starting template, were homogeneously monomeric in solution, and induced far lower receptor dimerization than rhG-CSF. Whereas 3 of the 4 tested designs showed residual receptor activation, 1 design (bop1) showed purely antagonistic effect, with no residual receptor activation. Upon inspecting the crystal structure of bop1, which matched the design model accurately, we assume for bop1 the receptor:ligand interactions mediated through secondary binding site are insufficient to overcome the thermodynamic cost of associating a strongly distorted 2:1 complex at the cell surface. Hence, we further improved bop1 by enhancing the affinity of its primary binding site through computational redesign and experimental screening. This led us to identify an improved variant (bop3), which bound G-CSFR approximately 10-fold tighter, outcompeted rhG-CSF-induced receptor assembly on HeLa cells, and was 3-fold stronger in inhibiting G-CSF-induced proliferation of NFS-60 cells. Finally, our in vitro assays show bop3 to be potent and specific in inhibiting G-CSF activity in an acute myeloid leukemia cell line and in primary hematopoietic stem cells.

Our approach to deploy a de novo-designed protein template to outcompete the native ligand precisely at its high-affinity binding site on the receptor surface bears several advantages. Among the advantages, is the guaranteed maximum epitope overlap between the inhibitor and the native ligand, ensuring effective inhibition while avoiding recourse to any epitope mapping procedure. Moreover, the biophysically superior features of the de novo-designed template gives room for substantial functional enhancement, where the latter often comes at the cost of the protein’s stability [70]. In this study, we started from our previous design Boskar4, which is thermally and proteolytically stable, and has maintained its stability after substantial redesign. Particularly, the most potent inhibitor bop3, which behaves as a strict monomer in solution, and binds G-CSFR 100-fold tighter than Boskar4, still preserves the hyper-thermostability of the parent template (Fig 5A and Fig O*A-B* in S1 Text). The high yield, solubility, and stability of bop3 greatly facilitate its further modification, production, and downstream processing, and obviate the need for cold chain handling.

Excessive G-CSFR signaling manifests in different pathologies depending on the type of the cells involved for which no targeted therapy is currently approved. Several hematopoietic malignancies of myeloid origin are strongly associated with the overexpression, acquired or inherited mutations of G-CSFR [19,71–75]. Our results on inhibiting G-CSF activity in a leukemia cell line demonstrate the therapeutic potential of our designs against myeloid malignancies. Furthermore, besides promoting leukemia, G-CSFR signaling can be responsible for the progression and metastasis of several tumors, and was shown as a potential target for treating glial [76,77], bladder [25], colon [21], ovary [22], and breast [23,24] cancers. Recently, some prevalent inflammatory conditions have been also tightly linked to elevated local or systemic levels of G-CSF. These include elevated G-CSF secretion in lung tissue in chronic obstructive pulmonary disease [78] or in peripheral blood of patients with rheumatoid arthritis [79], vasculitis [80], cystic fibrosis [81], and inflammatory bowel disease [82], where inhibition of G-CSFR was effective in reducing neutrophilic inflammation in animal models [83]. These all represent potential therapeutic applications where our presented designs can be advantageous over monoclonal antibodies in terms of ease of production, stability, and local bioavailability [84–86]. Our designed G-CSFR binders can also be used as homing or labeling tags, to increase the local targeting specificity of functional payloads to G-CSFR-expressing cells, without activating them. Given the stability of our designed helical bundles, and unlike immunoglobulin-based binders, their folding robustness provides for minimal folding interference with other fused proteins [87]. For example, bop2 as a compact bivalent activator of G-CSFR can be deployed as a fusion with another cytokine (i.e., as a *fusokine* [88]) to achieve co-targeting and co-activation of 2 or more receptors. Conversely, a binder such as bop3 can be readily used, in fusion with a different monovalent receptor binder, as a synthetic or surrogate cytokine [89,90] to induce novel receptor associations. These latter applications can greatly overcome the otherwise pleiotropic effects or systemic toxicities of cytokine therapies.

Finally, the strategy of simplifying a cytokine’s fold to derive non-activating variants can be readily extended to other helical cytokines that act on type I receptors. For example, simplifying the folds of thrombopoietin (TPO) and interleukin-11 (IL-11) has allowed us to obtain more stable binders against the thrombopoietin receptor (TPOR) and glycoprotein 130 (gp130), respectively (Figs Q and R in S1 Text). These simplified-fold designs clearly possess improved production and biophysical properties, which facilitate the enhancement of their specificity and affinity to their target receptor.

## Supporting information

S1 Text **Fig A. AlphaFold3 predicts the designs to induce unfavorable receptor orientations. (A–F)** AlphaFold3 predicts the expected parallel and antiparallel orientations induced by bop2 or boa2 indicate longer C-terminal spacing of the receptor subunits, and unfavored orientations with respect to membrane-embedded receptors. Representatives of the top 5 models are shown for bop (**A**) and boa (**D**). **(F–H)** In contrast, the 2:2 G-CSF:G-CSFR complex shows much more proximal inter-TMD spacing (Fig B in S1 Text). Shown are the first 2 models which represent the conformational diversity of the 5 predicted models. Prediction quality metrics for the structures (pLDDT: **B**, **D** and **H**; pAE: **C**, **F**, and **I**) are plotted next to the predicted structures. **Fig B. Inter-TMD spacing of the AlphaFold3 models of design:G-CSFR complexes predict a much larger inter-TMD spacing in comparison to the G-CSF:G-CSFR complex**. Boxplots of the spacing measured from the 5 models generated by each prediction. Numerical data for graphical items in this figure can be found in S1 Data. **Fig C. AlphaFold2 models predictions of the bifaceted designs and the bv6 template show minimal deviation from the template. (A)** Monomeric design models. **(B)** pLDDT values for the predicted structures. **Fig D. The G-CSFR-binding site in all design models aligned more poorly to the secondary binding site compared to the primary binding site**. AlphaFold2 models of the designed G-CSFR binders bop1, bop2, boa1, and boa2. The corresponding residues of the primary binding site and secondary binding site were structurally aligned to the corresponding residues of G-CSF (from PBD:2D9Q) to determine their backbone atoms RMSD. Numerical data for graphical items in this figure can be found in S1 Data. **Fig E. Oligomeric state of Boskar4 in solution dictates its effect on either activating or inhibiting G-CSFR. (A)** The illustration demonstrates how Boskar4 in a two-copy tandem connected by a short linker (Boskar4_st2) can dimerize the receptor subunits (left), while the monomeric fraction can inhibit G-CSF-induced receptor dimerization (right). **(B)** Analytical SEC shows the Boskar4 design to partition between a dimer and monomer in solution. Chromatography was performed using a Superdex 200 Increase 10/300 GL analytical column, where the data was obtained from [31]. **(C)** The monomeric fraction of Boskar4 could to some extent inhibit G-CSF-induced proliferation of NFS-60 cells. The figure shows the results of testing 10 μg/ml Boskar4 in a cell-based (NFS-60) competitive inhibition assay against 0 pg/ml, 100 pg/ml, and 1,000 pg/ml of G-CSF, where Boskar4 could outcompete 100 pg/ml G-CSF as indicated by an observed proliferation reduction. **(D)** Varying concentrations of Boskar4 titrated against 100 pg/ml G-CSF in the same cell assay explained also showed a clear reduction in proliferation at high concentration of monomeric Boskar4 (50 μg/ml). However, below non-inhibiting concentrations, an increase in proliferation was clear. Confluence values shown in (C, D) where obtained using the IncuCyte S3 Live-Cell Analysis System, where the bar plots show mean and standard deviation of 3 technical replicates. **(E)** Proliferative activity for non-inhibiting concentrations were also observed in NFS-60 proliferation assays for the affinity enhanced Boskar4 variant bv6 (dark gray circles). Shown is the fold change to untreated cells. The proliferation level of untreated cells is indicated by a gray box which represents mean and standard deviation of 12 parallel replicates. Numerical data for graphical items in this figure can be found in S1 Data. **Fig F**. **NanoDSF shows the bifaceted designs to be highly thermostable**. Heating (left column) and cooling (middle column) show no unfolding or refolding transitions for all 4 designs (bop1, bop2, boa1, and boa2) within the temperature range from 25 °C to 110 °C. Ratio values indicate relative fluorescence intensity at 350 nm and 330 nm. Scattering signal during the heating phase (right column) also indicate no aggregation of the designs. At least 4 (at most 7) technical replicates are shown for each design. Numerical data for graphical items in this figure can be found in S1 Data. **Fig G. SPR titrations of the bifaceted designs (bop/boa) compared to the starting template (bv6)**. SPR sensograms (black line) and the corresponding fit (green line) of a 2-fold dilution series starting at 20 nM of bv6, bop1, bop2, boa1, and boa2. Binding kinetic parameters that were calculated from the displayed fits (Table C in S1 Text; indicated in blue). Numerical data for graphical items in this figure can be found in S1 Data. **Fig H. Single molecule tracking of G-CSFR. (A)** Diffusion coefficients of G-CSFR under different treatments, as quantified by single-molecule tracking analysis on live HeLa cell membranes. **(B)** Receptor localization densities at the cell surface, indicating similar receptor expression of analyzed cells. Numerical data for graphical items in this figure can be found in S1 Data. **Fig I. bop2 induces proliferation only partial across a narrower concentration range when compared than G-CSF. (A)** Three biological replicas of NFS-60 proliferation assays performed under the comparable conditions using either rhG-CSF (circles) or bop2 (squares). Color shades indicate the experiments performed on the same place. The plot shows the individual measurements of the averaged values shown in Fig 2A. **(B)** *E*_*max*_ values for bop2 and rhG-CSF show the latter to be significantly more active. Numerical data for graphical items in Figs I and R*D-G* can be found in S1 Data. **Fig J. Tandemly repeating bop2 and boa2 yields agonists, with the parallel orientation induced by bop2 exhibiting higher cell activity**. (A) NFS-60 proliferation assays using short-tandems of bop2 (bop2_st2) or boa2 (boa2_st2). Mean and standard deviation of 3 independent replicates are shown as lines and shades, respectively. (B, C) Individual measurements of each construct fitted to derive the respective *EC*_*50*_ values (D), which show bop2_st2 to be significantly more active (compare Table C). Numerical data for graphical items in this figure can be found in S1 Data. **Fig K. The bop1 design is the most potently inhibits G-CSF activity with no residual receptor activation. (A)** The half-maximal effective concentration (*EC*_*50*_) of the bop2 was determined for non-inhibiting concentrations observed in NFS-60 activity assays (cf. Fig 2A). The fits of 3 independent experiments are shown. Mean and standard deviation values are provided in Table C. **(B)** The half-maximal inhibitory concentrations (*IC*_*50*_, indicated by dotted lines) were obtained from the corresponding competitive inhibition NFS-60 assay (cf. Fig 2B) by fitting the individual independent replicates. Mean and standard deviation of the obtained *IC*_*50*_ values is presented in Table C. Numerical data for graphical items in this figure can be found in S1 Data. **Fig L. Bop1 outcompetes G-CSF in a dose-dependent manner**. Competitive inhibition assays of NFS-60 proliferation using varying concentrations of bop1 against 3 concentrations of of G-CSF (200, 100, 50 pg/ml). The half-maximal inhibitory concentrations (*IC*_*50*_) were obtained from the corresponding fit. Numerical data for graphical items in this figure can be found in S1 Data. **Fig M. Library generation and screening. (A)** Illustration of the PCR mutagenesis strategy used for library generation. **(B)** Sanger sequencing analysis of mutated sites. **(C)** FACS enrichment of binding clones after the start and sort 1 to 5 (SSC-A vs. PE-A). **(D)** Single-variant screen Z-scores to identify top clones in 96-well assay format. Numerical data for graphical items in this figure can be found in S1 Data. **Fig N. The enhanced-affinity variants preserve the biophysical properties of the starting designs. (A)** SDS-PAGE of main fractions of enhanced-affinity variants bv21, bv22, bv23, and bv24 after preparative size exclusion chromatography. **(B)** CD analysis of the Boskar4 variants (bv6, bv8, bv21, bv22, bv23, and bv24) show them to be helical. **(C)** NanoDSF of the variants shows them be to hyper-thermostable. Folding stability and colloidal stability are represented by ratio of fluorescence intensity at 350 nm and 330 nm and the scattering signal, respectively, for at least 4 technical replicates. The raw image of S14A is provided in S1_raw_images and numerical data for graphical items in this figure can be found in S1 Data. **Fig O. The enhanced variants display stronger receptor-binding affinity and more potent inhibition of G-CSF-induced proliferation. (A–G)** SPR sensograms (black line) and the corresponding fits (green lines) of a 2-fold dilution series of the Boskar4 (A) and the enhanced Boskar4 variants bv6 (B), bv8 (C), bv21 (D), bv22 (E), bv23 (F), and bv24 (G). The highest concentrations measured were 1,250 nM for Boskar4 (B4), 125 nM for bv6 and bv8, and 31 nM for all other shown variants. Binding kinetic parameters derived from the displayed fits are listed in Table C (indicated in green). **(H)** Competitive inhibition NFS-60 assay with a constant concentration of 50 pg/ml rhG-CSF and the corresponding fit used to derive *IC*_*50*_ values for the designs (also compare Table C). Numerical data for graphical items in this figure can be found in S1 Data. **Fig P. Grafting the bv22 binding site as the primary binding site in bop1 yields the most potent inhibition, bop3. (A)** The bop3 is exclusively monomeric according to analytical SEC. Chromatogram shown was obtained using Cytiva Superdex 75 Increase 10/300 GL. **(B)** NanoDSF was performed in a temperature range from 25 °C to 110 °C. Fluorescence intensity ratio at 350 nm and 330 nm, and the scattering signal of 4 technical replicates are shown. **(C)** SPR sensograms (black lines) and the corresponding fits (green lines) of a 2-fold dilution series starting at 20 nM of bop1, bop3, and bv22. Binding kinetic parameters derived from these fits are provided in Table C (indicated in orange). **(D)** NFS-60 activity assay of 2 independent biological replicates of bop3 show no residual activity. The gray shade indicates the mean and standard deviation of the fold-change of untreated cells to the mean of untreated cells. **(E)** Competitive inhibition assays of G-CSF-induced proliferation in NFS-60 cells show bop3 to possess lower *IC*_*50*_ values (indicated by dotted vertical lines). Results were obtained from a fit of 3 independent replicates. Mean and standard deviation of the individual fits are provided in Table C. **(F)** Approximately 0.1 μg/ml of bop3 was tested in a healthy donor’s CD34+ HSPCs proliferation assay either without or with the addition of 1 ng/ml rhG-CSF, and 0.1 μg/ml Moevan_control (mvn_ctrl, [31]), a protein lacking a functional G-CSFR binding site, was employed as negative control. Shown is mean and standard deviation of 3 parallel replicates. Numerical data for graphical items in this figure can be found in S1 Data. **Fig Q. The design of refactored TPOR binders. (A)** Thrombopoietin (TPO) bridges 2 TPO receptor (TPOR) chains via site 1 (high-affinity; pink) and site 2 (low-affinity; gray) (PDB: 8G04) [91]. The up-up-down-down helical bundle of TPO features a beta-sheet that spans the long connectors. **(B)** The buto designs’ fold was simplified while preserving the high-affinity site and different circular permutations were tested. The AlphaFold2 model of the most active design, buto_a3, is shown. **(C)** buto_a3 shares minimal sequence similarity to TPO. **(D–F)** SPR sensograms and steady-state fitting of TPOR binding using bacterially expressed TPO **(D)**, buto_a3 **(E)**, and tandemly repeated buto_a3_t2 **(F)** results in affinities of 4.09 ± 1.84 μm, 10.56 ± 1.49 μm, and 0.88 ± 0.16 μm, respectively. Details of the design and experimental procedure for the TPOR binders can be found in S1 Methods. Numerical data for graphical items in this figure can be found in S1 Data. **Fig R. The design of refactored gp130 binding domains**. (A) Structure of Interleukin 11 (IL-11), an up-up-down-down bundle (PDB: 6O4O), and the design model of swift21_02 comprising a simplified up-down-up-down that preserve 1 gp130 binding site (site 2). (B, C) First generation design (swift21_02) showed larger surface area of exposed hydrophobic residues (residue polarity indicated by color scale; blue: polar, white: apolar) and a poorly packed core. Surface and core residues were computationally redesigned leading to swift4. (D, E) Swift4 design was monomeric and thermostable, as analyzed by SEC and CD measurements. (F, G) Swift4 binds gp130 with sub-micromolar affinity, which was 10-fold improved by tandem repetition (swift4_st2), aligning with the expected enhancement observed when comparing single and tandemly repeated domains. Details of the design and experimental procedures for the gp130 binder designs can be found in S1 Methods. Numerical data for graphical items in this figure can be found in S1 Data. **Table A**. Shown are the mutations in bv6 compared to the given design (bop1, bop2, boa1, and boa2) and the corresponding residues in G-CSF (2D9Q). The residues highlighted in red represent the additional mutations between corresponding designs with small and large secondary binding sites (e.g., bop1 and bop2). **Table B**. Sequences of constructs presented in this study. Boldface marks residues belonging to (primary: purple, secondary: black) binding sites. Italic marks residues representing additional mutations between corresponding designs with small and large secondary binding sites (e.g., bop1 and bop2). **Table C**. The estimated dissociation rate (*k*_*d*_), association rate (*k*_*a*_), and dissociation constant (*K*_*D*_) of the corresponding designs are provided the corresponding colors signify whether the samples were measured in the same experiments. SPR parameter for Boskar4 and bv6 colored in green were adopted from [34]. Furthermore, the half-maximal inhibitory concentration (*IC*_*50*_) and the half-maximal effective concentration (*EC*_*50*_) of NFS-60 assays are presented, along with indications of residual activity for non-inhibiting concentrations. **Table D**. Crystallographic Data Collection and Refinement Statistics.
(DOCX)

S1 Data Numerical data for graphical items in Figs 1D, 1E, 1G, 2A–D, 4C, 4E, 5A–H and Figs B, D, E*C-E*, F, G, H*A-B*, I*A-B*, J*A-D*, K*A-B*, L, M*B-D*, N*B-C*, O*A-H*, P*A-F*, Q*D-F*, and R*D-G* can be found in S1 Data. (XLSX)

S1 Raw Images The raw image of panel *A* of Fig N in S1 Text is provided in S1 Raw Images. (PDF)

S1 Methods Supplementary Methods. (DOCX)

## Abbreviations

AML: acute myeloid leukemia
CD: circular dichroism
FACS: fluorescence-activated cell sorting
G-CSFR: granulocyte-colony stimulating factor receptor
HSPC: hematopoietic stem and progenitor cell
IMAC: immobilized metal affinity chromatography
nanoDSF: nanoscale-differential scanning fluorimetry
RMSD: root mean square deviation
RT: room temperature
SEC: size exclusion chromatography
SLS: Swiss Light Source
TIRF: total internal reflection fluorescence
