# Supplementary material for: A strategy to design protein-based antagonists against type I cytokine receptors
Source: PLoS Biol. 2024 Nov 26;22(11):e3002883. doi: 10.1371/journal.pbio.3002883 (PMC11596305; doi:10.1371/journal.pbio.3002883)
Supplement: S1 Text — Fig A. AlphaFold3 predicts the designs to induce unfavorable receptor orientations. (A–F) AlphaFold3 predicts the expected parallel and antiparallel orientations induced by bop2 or boa2 indicate longer C-terminal spacing of the receptor subunits, and unfavored orientations with respect to membrane-embedded receptors. Representatives of the top 5 models are shown for bop (A) and boa (D). (F–H) In contrast, the 2:2 G-CSF:G-CSFR complex shows much more proximal inter-TMD spacing (Fig B in S1 Text). Shown are the first 2 models which represent the conformational diversity of the 5 predicted models. Prediction quality metrics for the structures (pLDDT: B, D and H; pAE: C, F, and I) are plotted next to the predicted structures. Fig B. Inter-TMD spacing of the AlphaFold3 models of design:G-CSFR complexes predict a much larger inter-TMD spacing in comparison to the G-CSF:G-CSFR complex. Boxplots of the spacing measured from the 5 models generated by each prediction. Numerical data for graphical items in this figure can be found in S1 Data. Fig C. AlphaFold2 models predictions of the bifaceted designs and the bv6 template show minimal deviation from the template. (A) Monomeric design models. (B) pLDDT values for the predicted structures. Fig D. The G-CSFR-binding site in all design models aligned more poorly to the secondary binding site compared to the primary binding site. AlphaFold2 models of the designed G-CSFR binders bop1, bop2, boa1, and boa2. The corresponding residues of the primary binding site and secondary binding site were structurally aligned to the corresponding residues of G-CSF (from PBD:2D9Q) to determine their backbone atoms RMSD. Numerical data for graphical items in this figure can be found in S1 Data. Fig E. Oligomeric state of Boskar4 in solution dictates its effect on either activating or inhibiting G-CSFR. (A) The illustration demonstrates how Boskar4 in a two-copy tandem connected by a short linker (Boskar4_st2) can dimerize the receptor subunits (l [file pbio.3002883.s001.docx]

Supplementary material

A Strategy to Design Protein-based Antagonists Against Type I Cytokine Receptors

Timo Ullrich^1^, Olga Klimenkova^2,†^, Christoph Pollmann^3,†^, Asma Lasram^3^, Valeriia Hatskovska^1,2^, Kateryna Maksymenko^1^, Matej Milijaš-Jotić^1^, Lukas Schenk^2^, Claudia Lengerke^2^, Marcus D. Hartmann^1,4^, Jacob Piehler^3^, Julia Skokowa^2^, Mohammad ElGamacy^1,2*^

^1^ Max Planck Institute for Biology, Department of Protein Evolution, Tübingen, Germany

^2^ Translational Oncology, Internal Medicine II, University Hospital Tübingen, Germany

^3^ Department of Biology/Chemistry and Center for Cellular Nanoanalytics, Osnabrück University, 49076 Osnabrück, Germany

^4^ Interfaculty Institute of Biochemistry, University of Tübingen, Germany

^†^ equally-contributing authors

^*^ corresponding author: mohammad.elgamacy@med.uni-tuebingen.de

**Supplementary figures A-R.**

**Supplementary tables A-D.**

# ***Supplementary Figures***


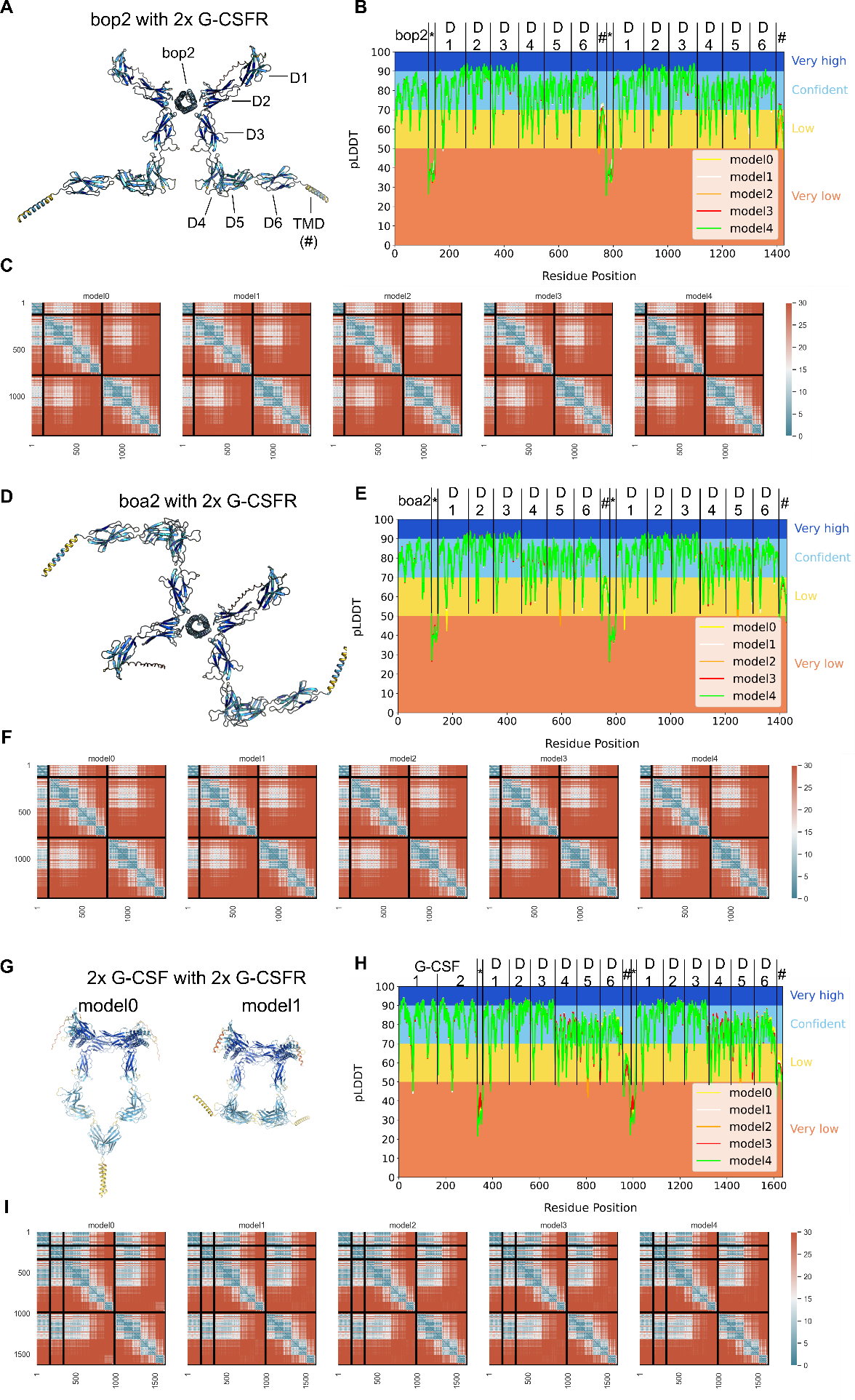


**Figure A. AlphaFold3 predicts the designs to induce unfavorable receptor orientations. (A-F)** AlphaFold3 [1] predicts the expected parallel and antiparallel orientations induced by bop2 or boa2 indicate longer C-terminal spacing of the receptor subunits, and unfavored orientations with respect to membrane-embedded receptors. Representatives of the top five models are shown for bop (**A**) and boa (**D**). **(F-H)** In contrast, the 2:2 G-CSF:G-CSFR complex shows much more proximal inter-TMD spacing (Fig B). Shown are the first two models which represent the conformational diversity of the five predicted models. Prediction quality metrics for the structures (pLDDT: **B**, **D** and **H**; pAE: **C**, **F**, and **I**) are plotted next to the predicted structures.


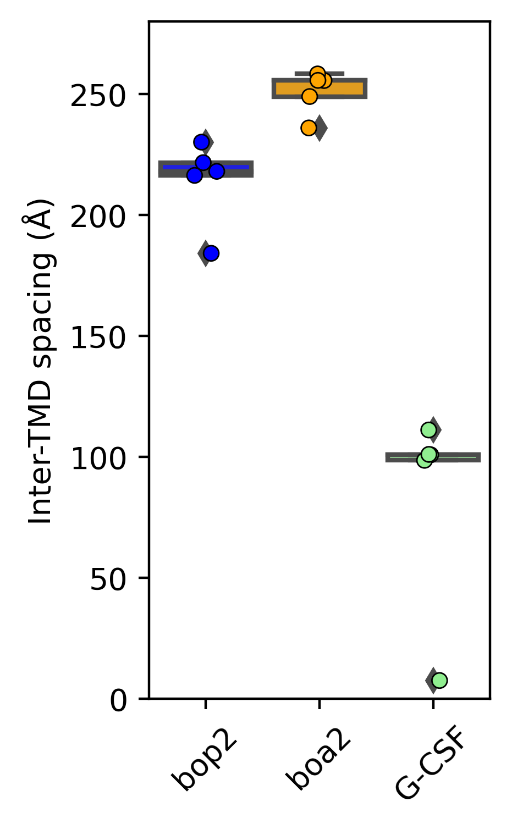


**Figure B. Inter-TMD spacing of the AlphaFold3 models of design:G-CSFR complexes predict a much larger inter-TMD spacing in comparison to the G-CSF:G-CSFR complex.** Boxplots of the spacing measured from the five models generated by each prediction. Numerical data for graphical items in this Figure can be found in S1 Data.


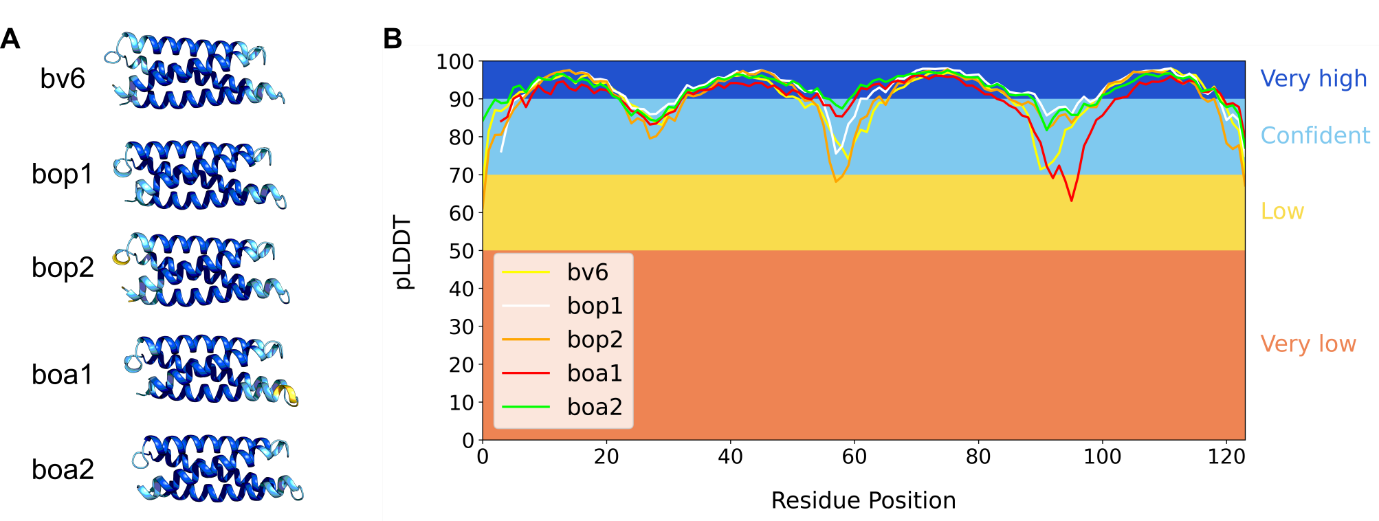


**Figure C. AlphaFold2 [2] models predictions of the bifaceted designs and the bv6 template show minimal deviation from the template. (A)** Monomeric design models. **(B)** pLDDT values for the predicted structures.


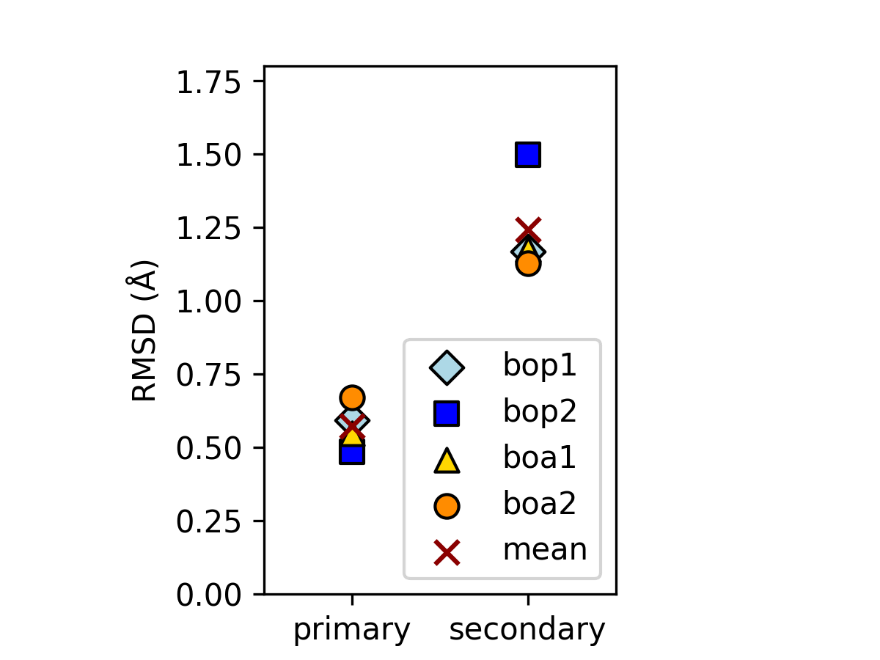


**Figure D. The G-CSFR-binding site in all design models aligned more poorly to the secondary binding site compared to the primary binding site.** AlphaFold2 models of the designed G-CSFR binders bop1, bop2, boa1 and boa2. The corresponding residues of the primary binding site and secondary binding site were structurally aligned to the corresponding residues of G-CSF (from PBD:2D9Q) to determine their backbone atoms RMSD. Numerical data for graphical items in this Figure can be found in S1 Data.


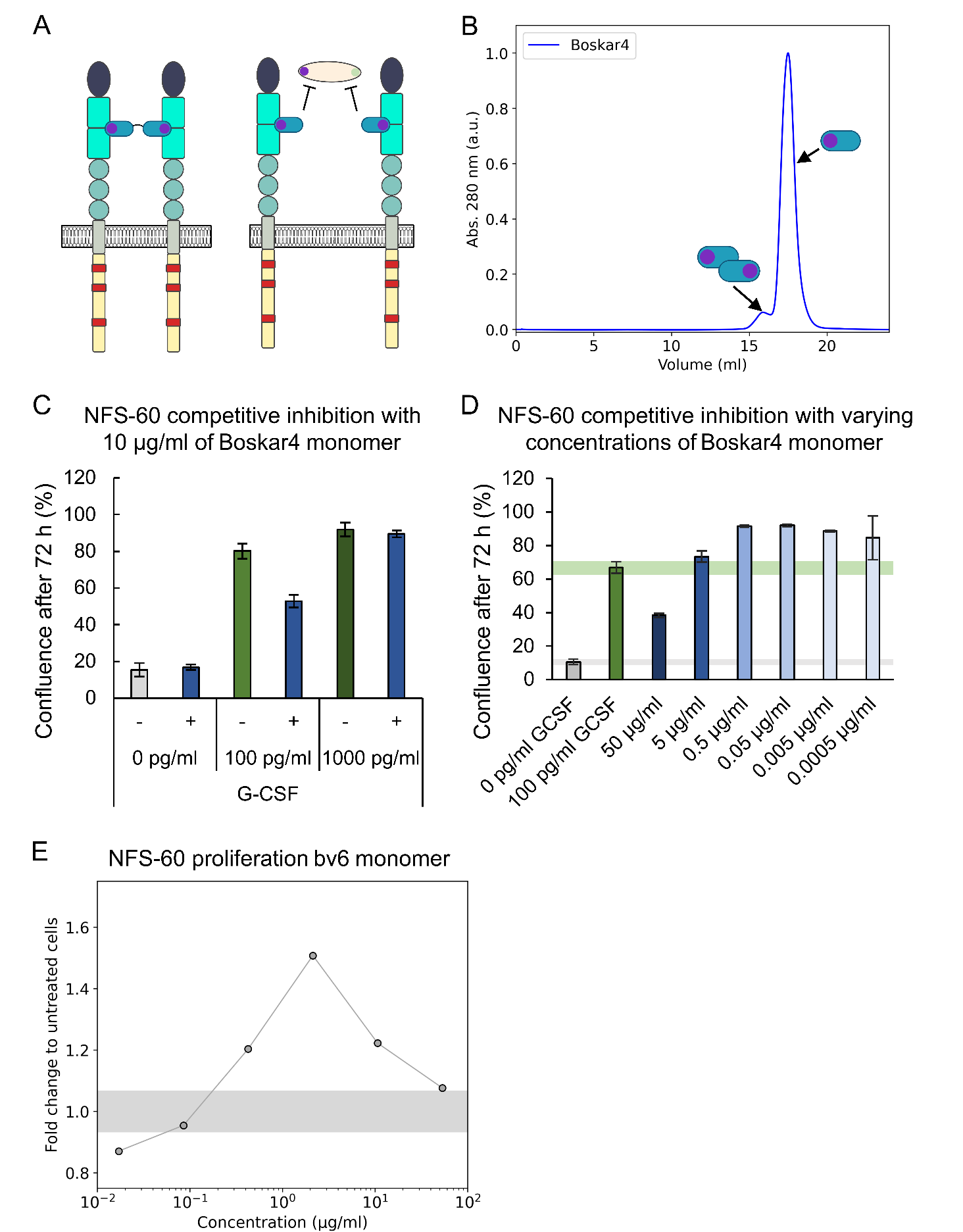


**Figure E. Oligomeric state of Boskar4 in solution dictates its effect on either activating or inhibiting G-CSFR. (A)** The illustration demonstrates how Boskar4 in a two-copy tandem connected by a short linker (Boskar4_st2) can dimerize the receptor subunits (left), while the monomeric fraction can inhibit G-CSF-induced receptor dimerization (right). **(B)** Analytical SEC shows the Boskar4 design to partition between a dimer and monomer in solution. Chromatography was performed using a Superdex 200 Increase 10/300 GL analytical column, where the data was obtained from [3]. **(C)** The monomeric fraction of Boskar4 could to some extent inhibit G-CSF-induced proliferation of NFS-60 cells. The figure shows the results of testing 10 µg/mL Boskar4 in a cell-based (NFS-60) competitive inhibition assay against 0 pg/mL, 100 pg/mL and 1000 pg/mL of G-CSF, where Boskar4 could outcompete 100 pg/mL G-CSF as indicated by an observed proliferation reduction. **(D)** Varying concentrations of Boskar4 titrated against 100 pg/mL G-CSF in the same cell assay explained also showed a clear reduction in proliferation at high concentration of monomeric Boskar4 (50 µg/mL). However below non-inhibiting concentrations, an increase in proliferation was clear. Confluence values shown in (C, D) where obtained using the IncuCyte S3 Live-Cell Analysis System, where the bar plots show mean and standard deviation of three technical replicates. **(E)** Proliferative activity for non-inhibiting concentrations were also observed in NFS-60 proliferation assays for the affinity enhanced Boskar4 variant bv6 (dark gray circles). Shown is the fold change to untreated cells. The proliferation level of untreated cells is indicated by a gray box which represents mean and standard deviation of twelve parallel replicates. Numerical data for graphical items in this Figure can be found in S1 Data.


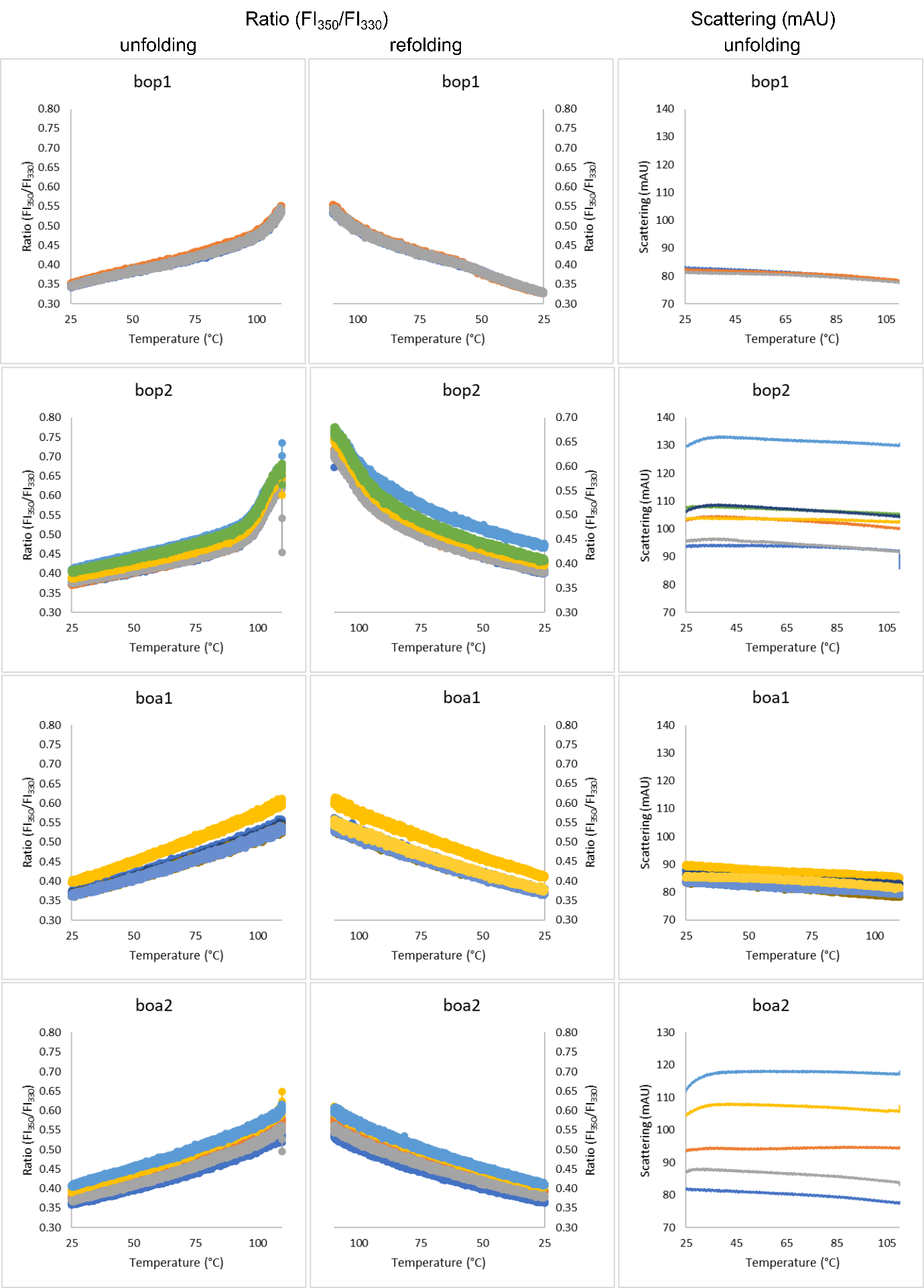


**Figure F**. **NanoDSF shows the bifaceted designs to be highly thermostable.** Heating (left column) and cooling (middle column) show no unfolding or refolding transitions for all four designs (bop1, bop2, boa1, and boa2) within the temperature range from 25 °C to 110 °C. Ratio values indicate relative fluorescence intensity at 350 nm and 330 nm. Scattering signal during the heating phase (right column) also indicate no aggregation of the designs. At least four (at most seven) technical replicates are shown for each design. Numerical data for graphical items in this Figure can be found in S1 Data.


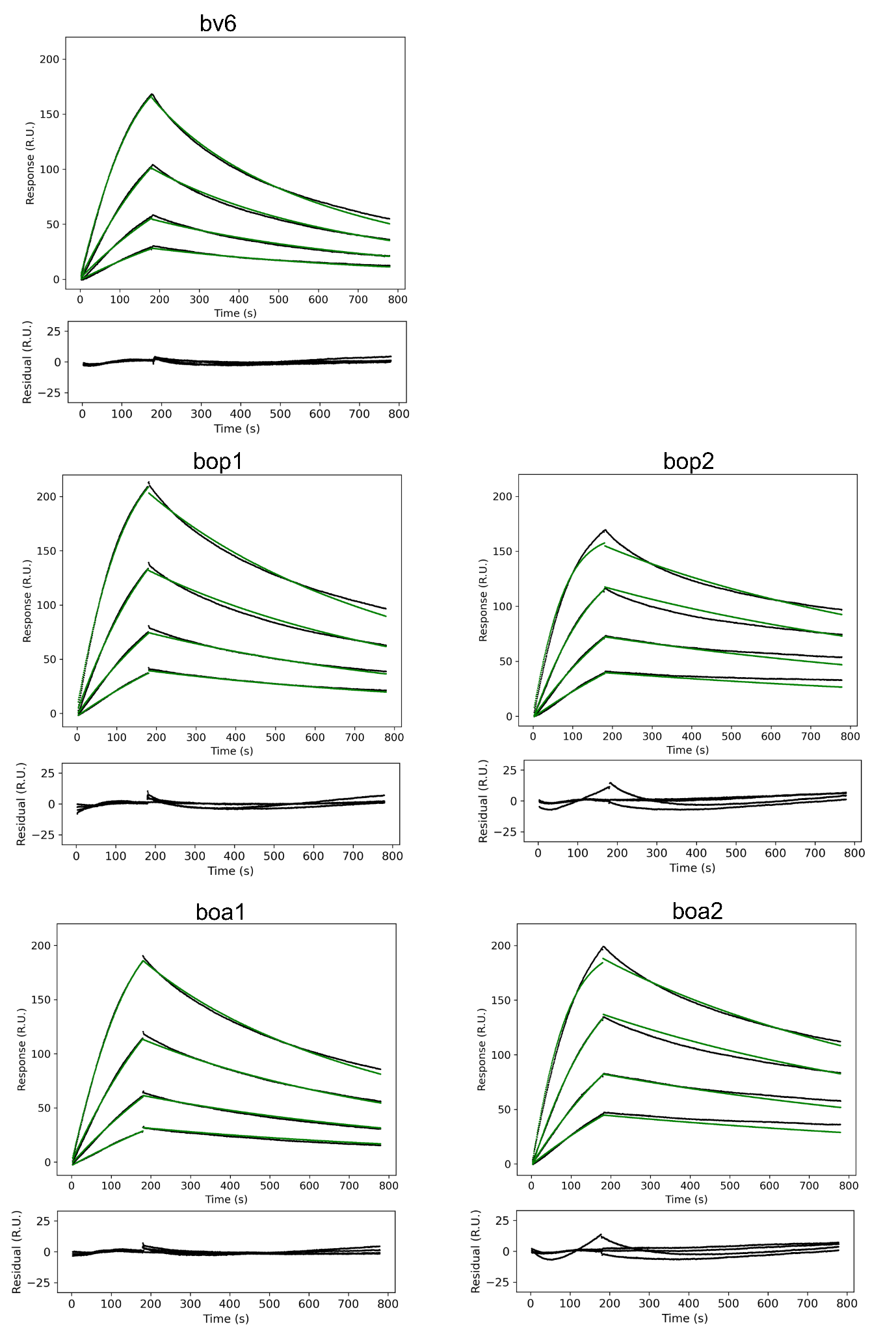


**Figure G. SPR titrations of the bifaceted designs (bop/boa) compared to the starting template (bv6).** SPR sensograms (black line) and the corresponding fit (green line) of a two-fold dilution series starting at 20 nM of bv6, bop1, bop2, boa1, and boa2. Binding kinetic parameters that were calculated from the displayed fits (Table C; indicated in blue). Numerical data for graphical items in this Figure can be found in S1 Data.


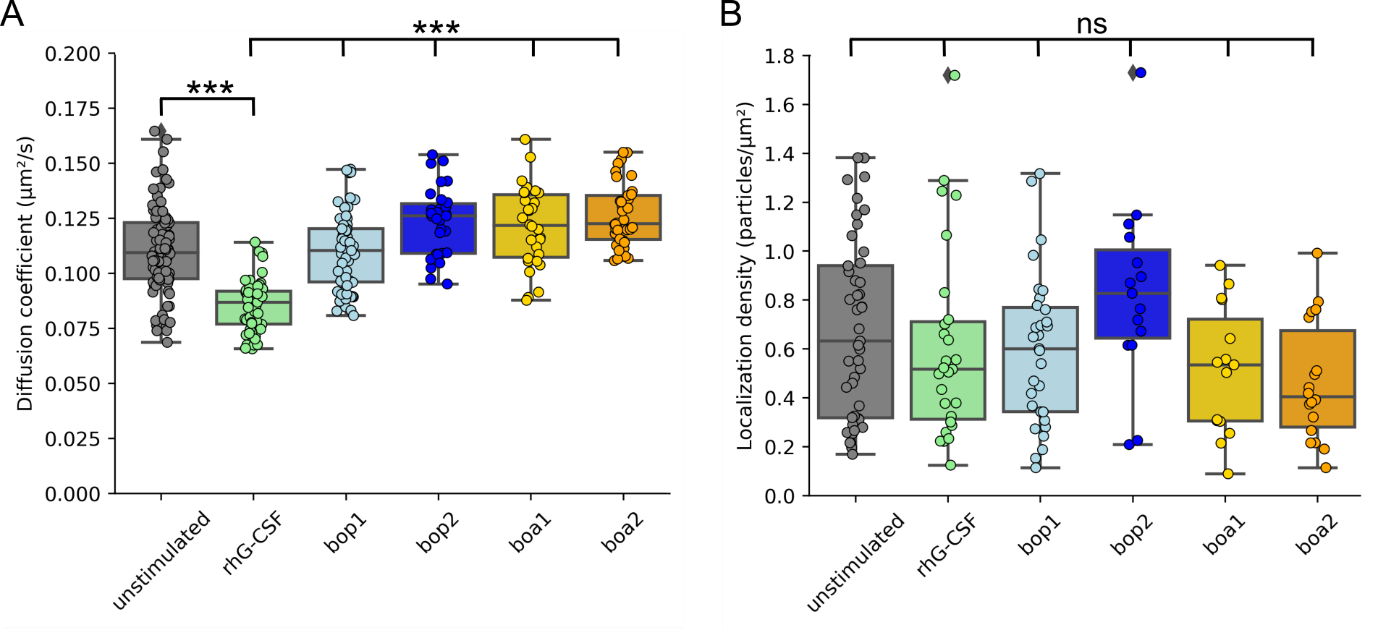


**Figure H. Single molecule tracking of G-CSFR (A)** Diffusion coefficients of G-CSFR under different treatments, as quantified by single-molecule tracking analysis on live HeLa cell membranes. **(B)** Receptor localization densities at the cell surface, indicating similar receptor expression of analyzed cells. Numerical data for graphical items in this Figure can be found in S1 Data.


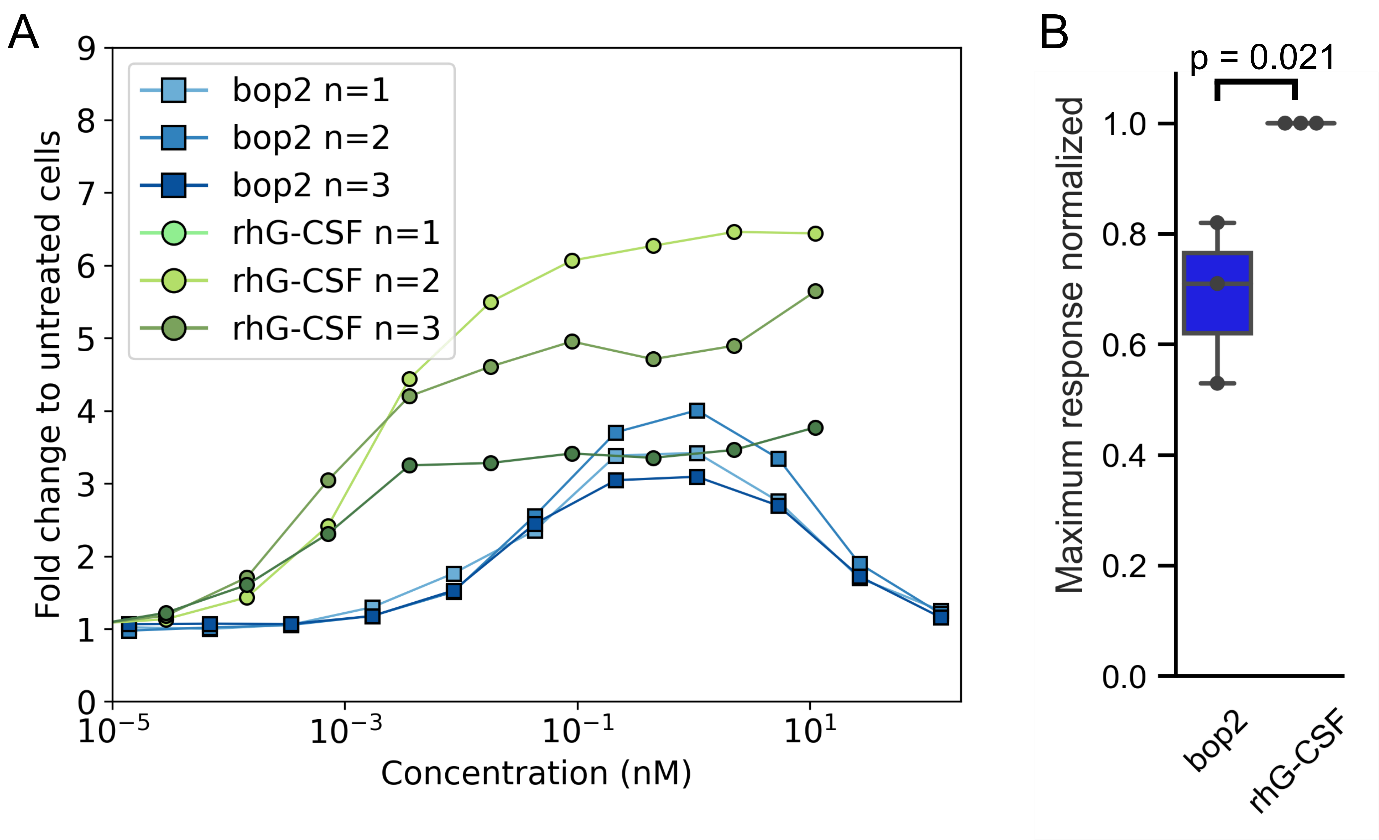


**Figure I. bop2 induces proliferation only partial across a narrower concentration range when compared than G-CSF. (A)** Three biological replicas of NFS-60 proliferation assays performed under the comparable conditions using either rhG-CSF (circles) or bop2 (squares). Color shades indicate the experiments performed on the same place. The plot shows the individual measurements of the averaged values shown in Fig. 2A. **(B)** *E_max_* values for bop2 and rhG-CSF show the latter to be significantly more active. Data for cell activity of rhG-CSF was taken from [4]. Numerical data for graphical items in Figs I and R*D-G* be found in S1 Data.


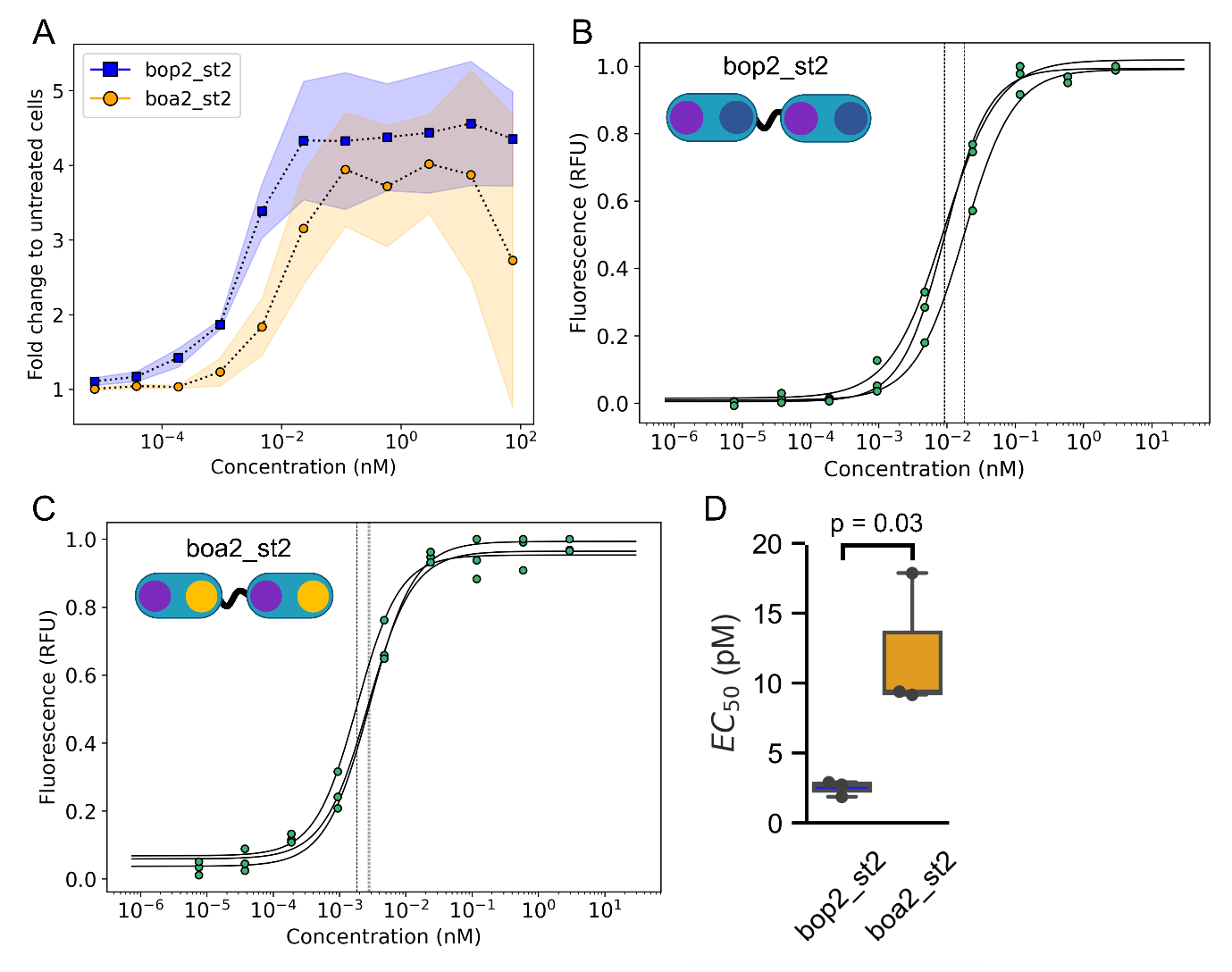


**Figure J. Tandemly repeating bop2 and boa2 yields agonists, with the parallel orientation induced by bop2 exhibiting higher cell activity.** (A) NFS-60 proliferation assays using short-tandems of bop2 (bop2_st2) or boa2 (boa2_st2). Mean and standard deviation of three independent replicates are shown as lines and shades, respectively. (B, C) individual measurements of each construct fitted to derive the respective *EC_50_* values (D), which show bop2_st2 to be significantly more active (compare Table C). Numerical data for graphical items in in this Figure can be found in S1 Data.


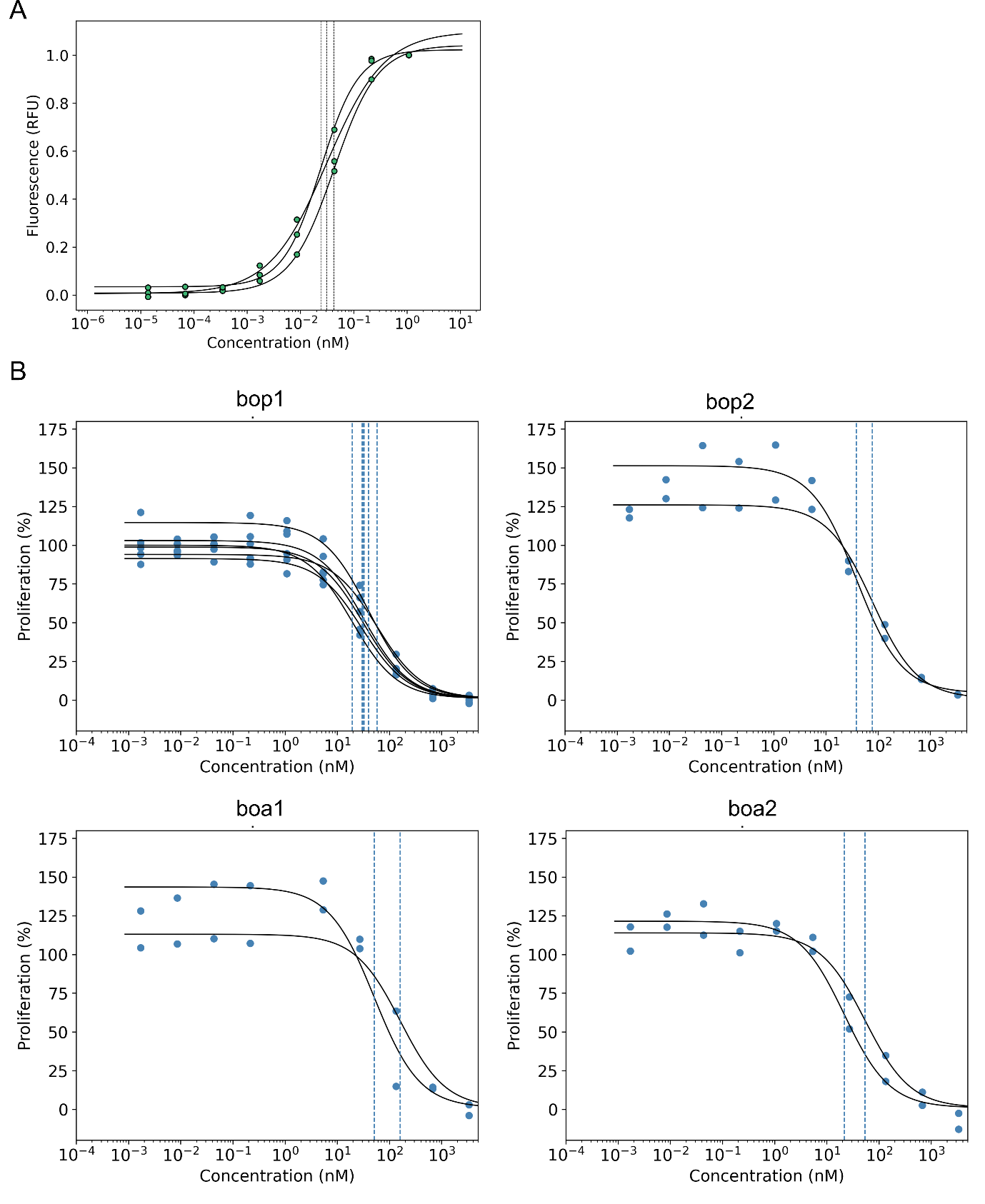


**Figure K. The bop1 design most potently inhibits G-CSF activity with no residual receptor activation. (A)** The half-maximal effective concentration (*EC_50_*) of the bop2 was determined for non-inhibiting concentrations observed in NFS-60 activity assays (cf. Fig 2A). The fits of three independent experiments are shown. Mean and standard deviation values are provided in Table C. **(B)** The half-maximal inhibitory concentrations (*IC_50_*, indicated by dotted lines) were obtained from the corresponding competitive inhibition NFS-60 assay (cf. Fig 2B) by fitting the individual independent replicates. Mean and standard deviation of the obtained *IC_50_* values is presented in Table C. Numerical data for graphical items in this Figure can be found in S1 Data.


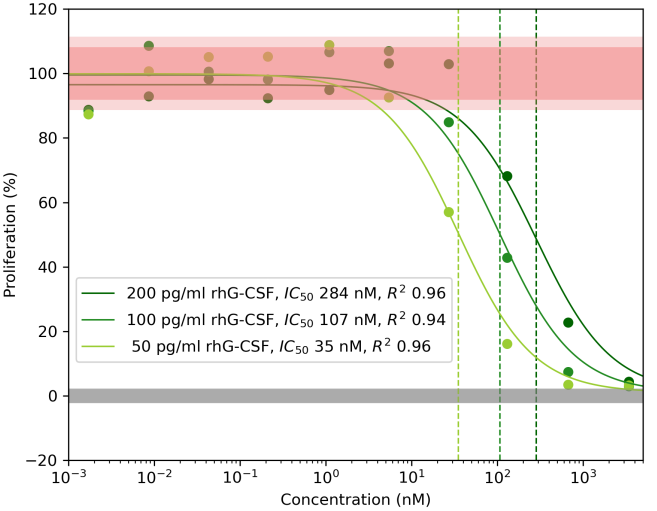


**Figure L. Bop1 outcompetes G-CSF in a dose-dependent manner.** Competitive inhibition assays of NFS-60 proliferation using varying concentrations of bop1 against three concentrations of of G-CSF (200, 100, 50 pg/mL). The half-maximal inhibitory concentrations (*IC_50_*) were obtained from the corresponding fit. Numerical data for graphical items in this Figure can be found in S1 Data.


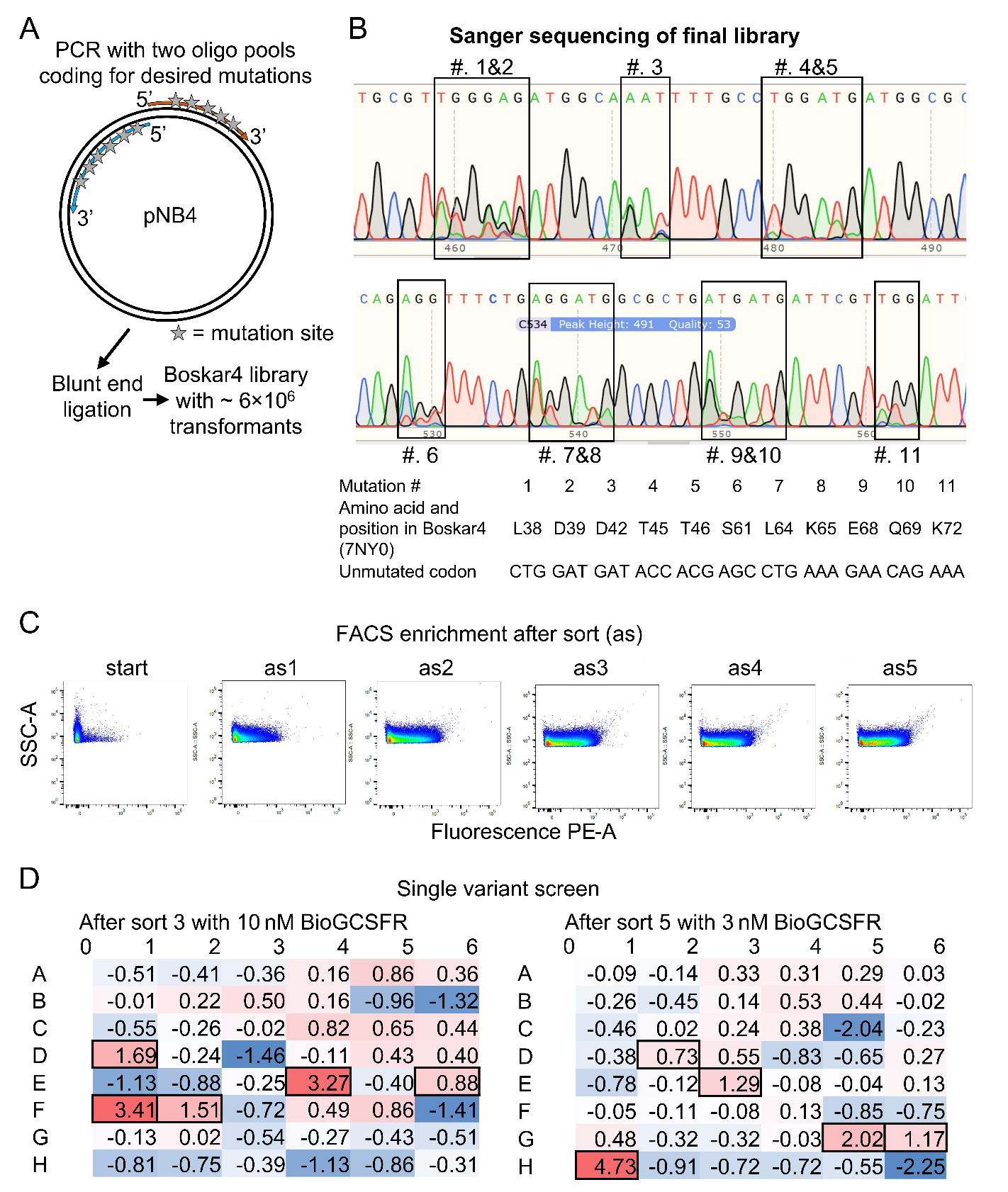


**Figure M. Library generation and screening.** **(A)** Illustration of the PCR mutagenesis strategy used for library generation. **(B)** Sanger sequencing analysis of mutated sites. **(C)** FACS enrichment of binding clones after the start and sort 1 to 5 (SSC-A vs. PE-A). **(D)** Single-variant screen Z-scores to identify top clones in 96-well assay format. Numerical data for graphical items in this Figure can be found in S1 Data.


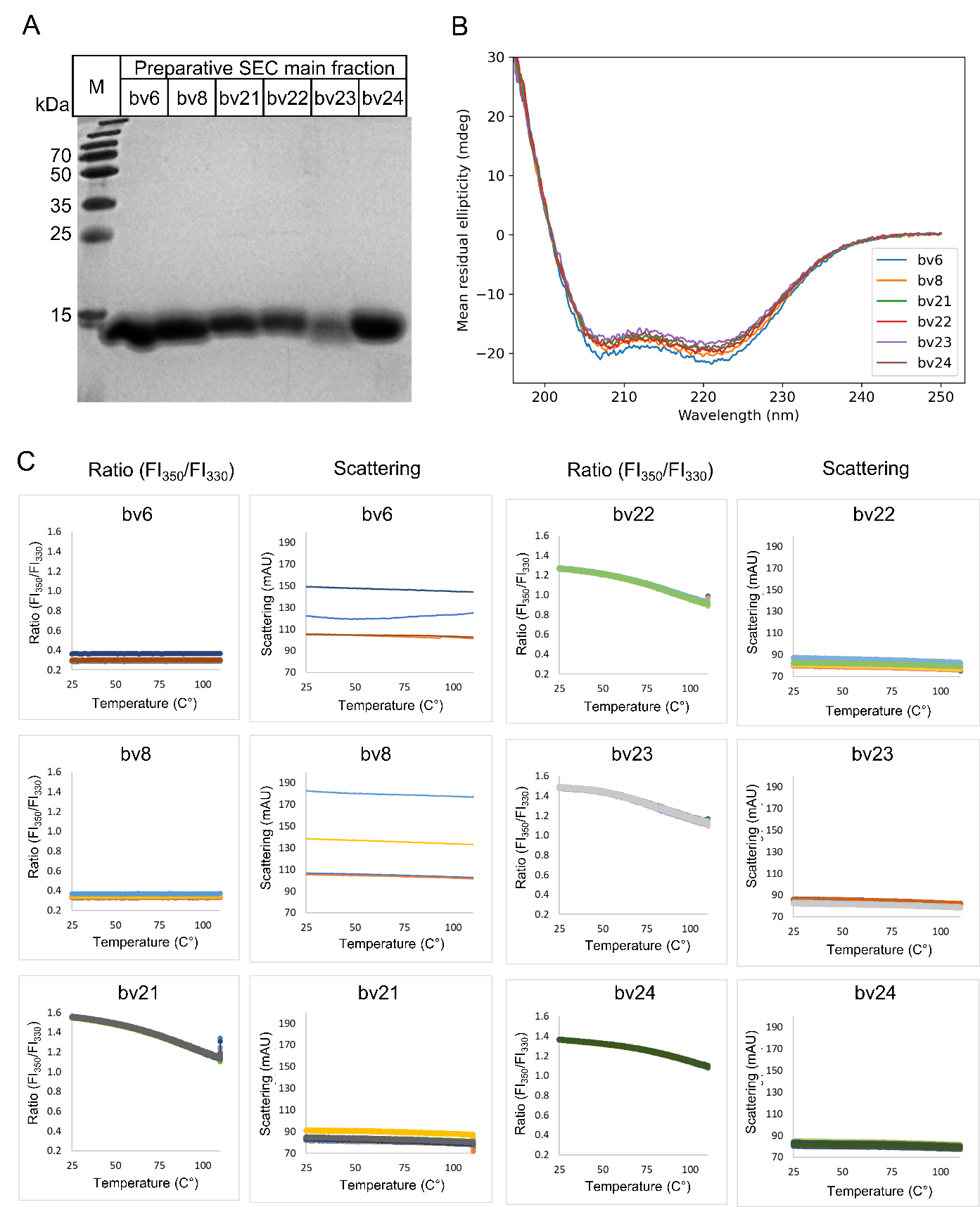


**Figure N. The enhanced-affinity variants preserve the biophysical properties of the starting designs. (A)** SDS-PAGE of main fractions of enhanced-affinity variants bv21, bv22, bv23, and bv24 after preparative size exclusion chromatography. **(B)** CD analysis of the Boskar4 variants (bv6, bv8, bv21, bv22, bv23 and bv24) show them to be helical. **(C)** NanoDSF of the variants shows them be to hyper-thermostable. Folding stability and colloidal stability are represented by ratio of fluorescence intensity at 350 nm and 330 nm and the scattering signal, respectively, for at least 4 technical replicates. The raw image of panel *A* is provided in S1 Raw Images and numerical data for graphical items in this Figure can be found in S1 Data.


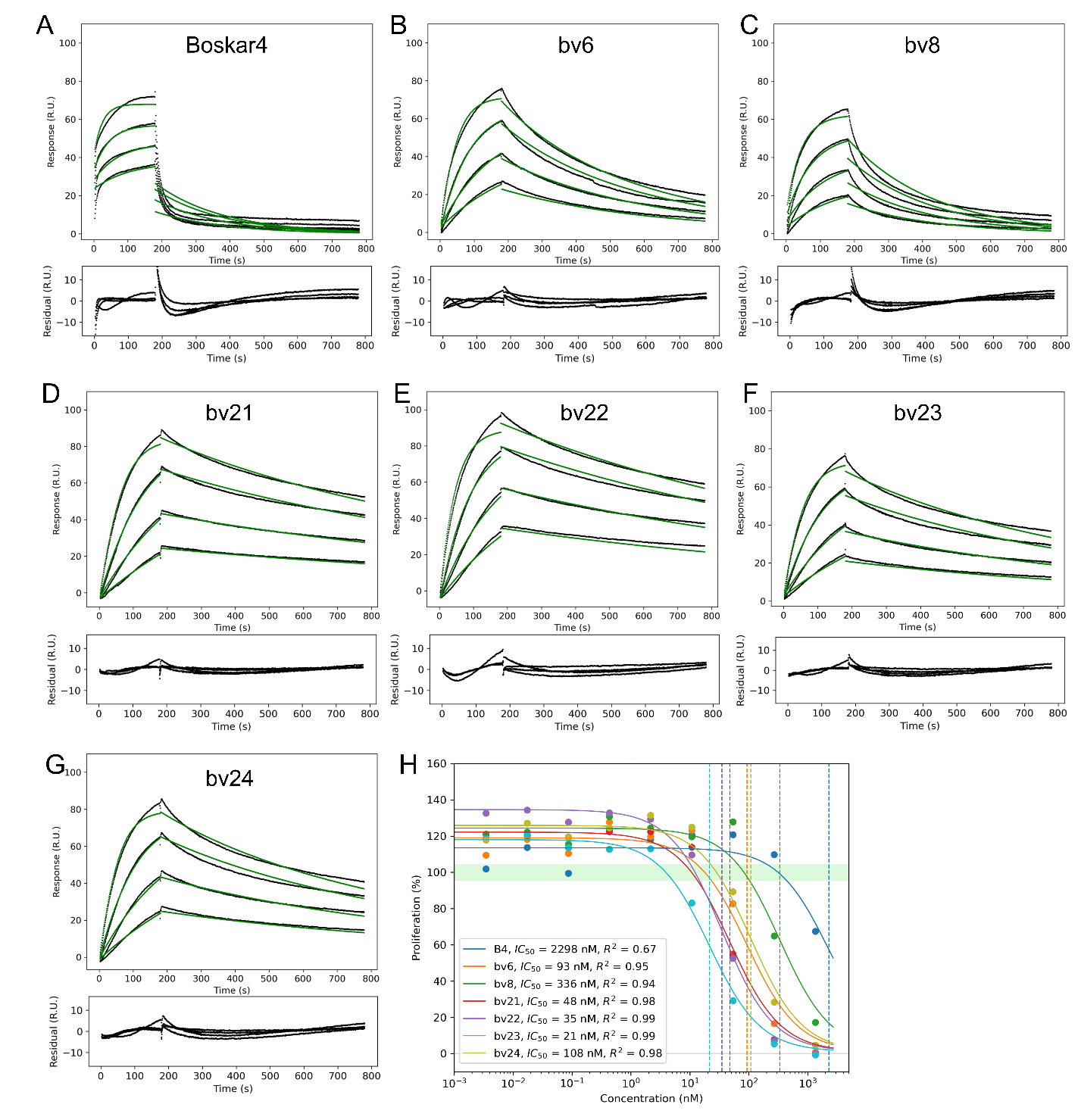


**Figure O. The enhanced variants display stronger receptor-binding affinity and more potent inhibition of G-CSF-induced proliferation. (A-G)** SPR sensograms (black line) and the corresponding fits (green lines) of a two-fold dilution series of the Boskar4 (A) and the enhanced Boskar4 variants bv6 (B), bv8 (C), bv21 (D), bv22 (E), bv23 (F) and bv24 (G). The highest concentrations measured were 1250 nM for Boskar4 (B4), 125 nM for bv6 and bv8 and 31 nM for all other shown variants. Binding kinetic parameters derived from the displayed fits are listed in Table C (indicated in green). **(H)** Competitive inhibition NFS-60 assay with a constant concentration of 50 pg/ml rhG-CSF and the corresponding fit used to derive *IC_50_* values for the designs (also compare Table C). Numerical data for graphical items in this Figure be found in S1 Data.


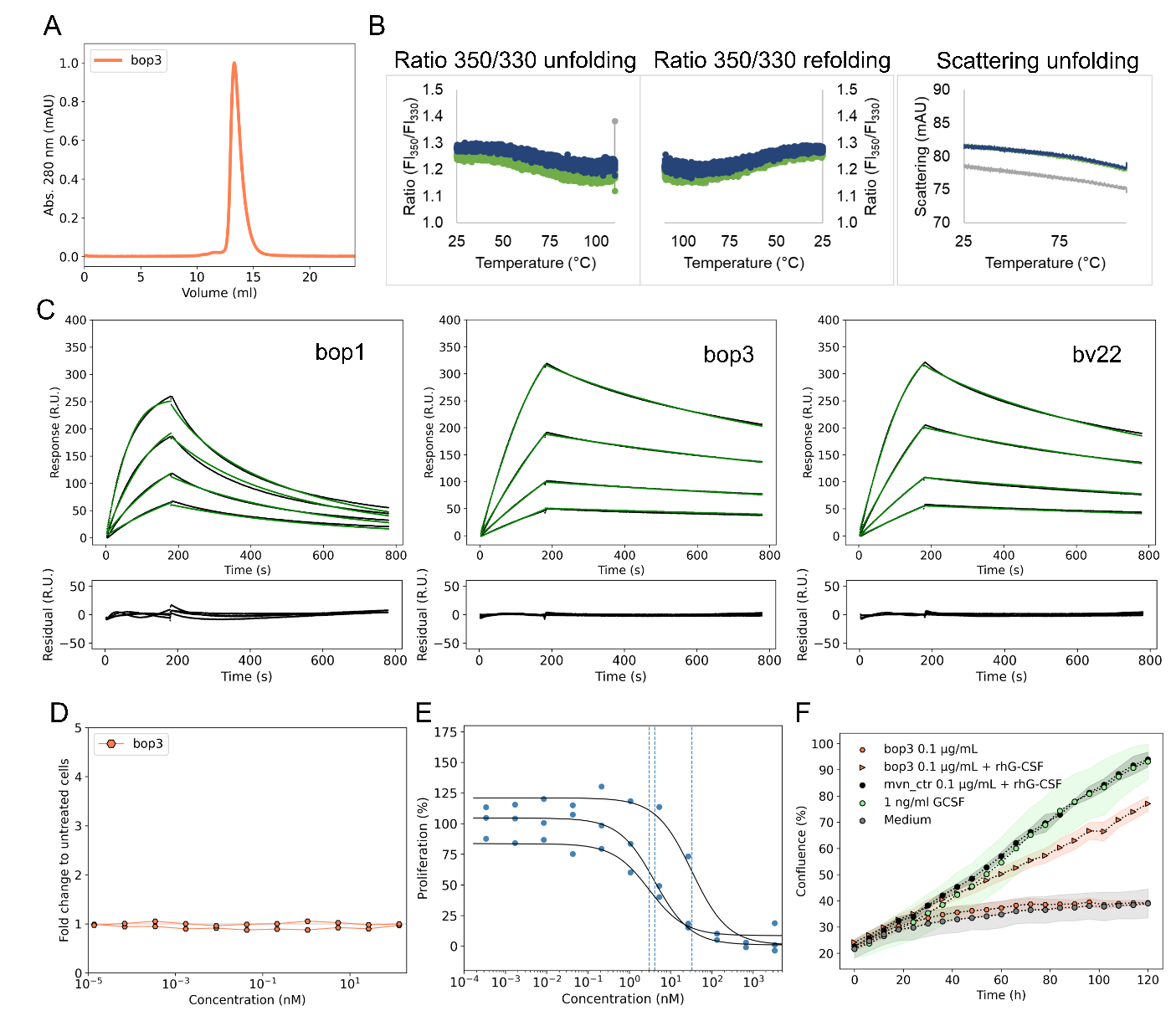


**Figure P. Grafting the bv22 binding site as the primary binding site in bop1 yields the most potent inhibition, bop3.** **(A)** The bop3 is exclusively monomeric according to analytical SEC. Chromatogram shown was obtained using Cytiva Superdex 75 Increase 10/300 GL. **(B)** NanoDSF was performed in a temperature range from 25 °C to 110 °C. fluorescence intensity ratio at 350 nm and 330 nm, and the scattering signal of 4 technical replicates are shown. **(C)** SPR sensograms (black lines) and the corresponding fits (green lines) of a two-fold dilution series starting at 20 nM of bop1, bop3 and bv22. Binding kinetic parameters derived from these fits are provided in Table C (indicated in orange). **(D)** NFS-60 activity assay of two independent biological replicates of bop3 show no residual activity. The gray shade indicates the mean and standard deviation of the fold-change of untreated cells to the mean of untreated cells. **(E)** Competitive inhibition assays of G-CSF-induced proliferation in NFS-60 cells show bop3 to possess lower *IC_50_* values (indicated by dotted vertical lines). Results were obtained from a fit of three independent replicates. Mean and standard deviation of the individual fits are provided in Table C. **(F)** 0.1 µg/ml of bop3 was tested in a healthy donor´s CD34+ HSPCs proliferation assay either without or with the addition of 1 ng/ml rhG-CSF. 0.1 µg/ml Moevan_control (mvn_ctrl, [3]), a protein lacking a functional G-CSFR binding site, was employed as negative control. Shown is mean and standard deviation of three parallel replicates. Numerical data for graphical items in this Figure can be found in S1 Data.


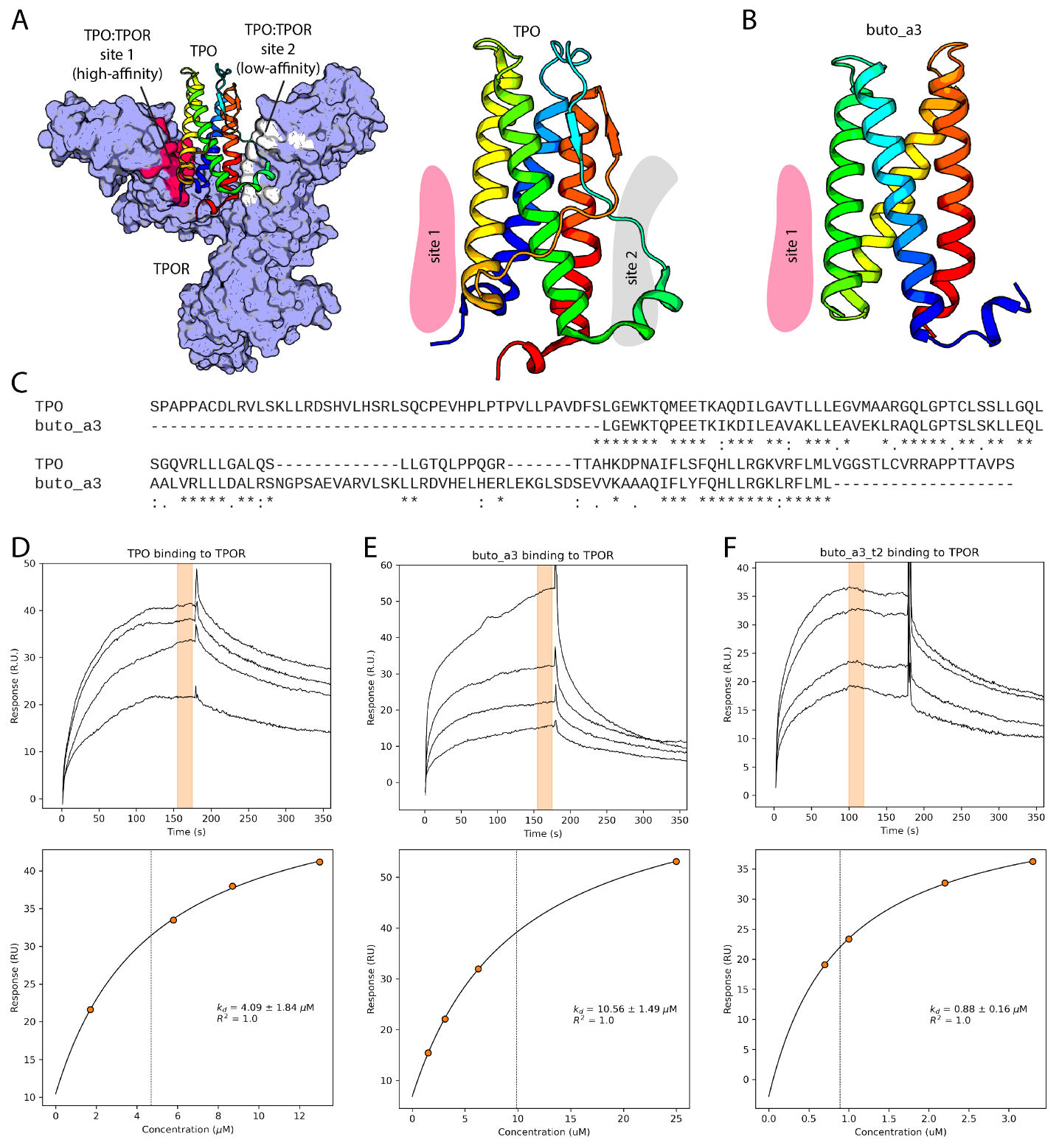


**Figure Q. The design of refactored TPOR binders.** **(A)** Thrombopoietin (TPO) bridges two TPO receptor (TPOR) chains via site 1 (high-affinity; pink) and site 2 (low-affinity; grey) (PDB: 8G04 [5]). The up-up-down-down helical bundle of TPO features a beta-sheet that spans the long connectors. **(B)** The buto designs’ fold was simplified while preserving the high-affinity site and different circular permutations were tested. The AlphaFold2 model of the most active design, buto_a3, is shown. **(C)** buto_a3 shares minimal sequence similarity to TPO. **(D-F)** SPR sensograms and steady state fitting of TPOR binding using bacterially-expressed TPO **(D)**, buto_a3 **(E)**, and tandemly repeated buto_a3_t2 **(F)** results in affinities of 4.09 ± 1.84 μM, 10.56 ± 1.49 μM, and 0.88 ± 0.16 μM, respectively. Details of the design and experimental procedure for the TPOR binder designs can be found in the S1 Methods. Numerical data for graphical items in this Figure can be found in S1 Data.


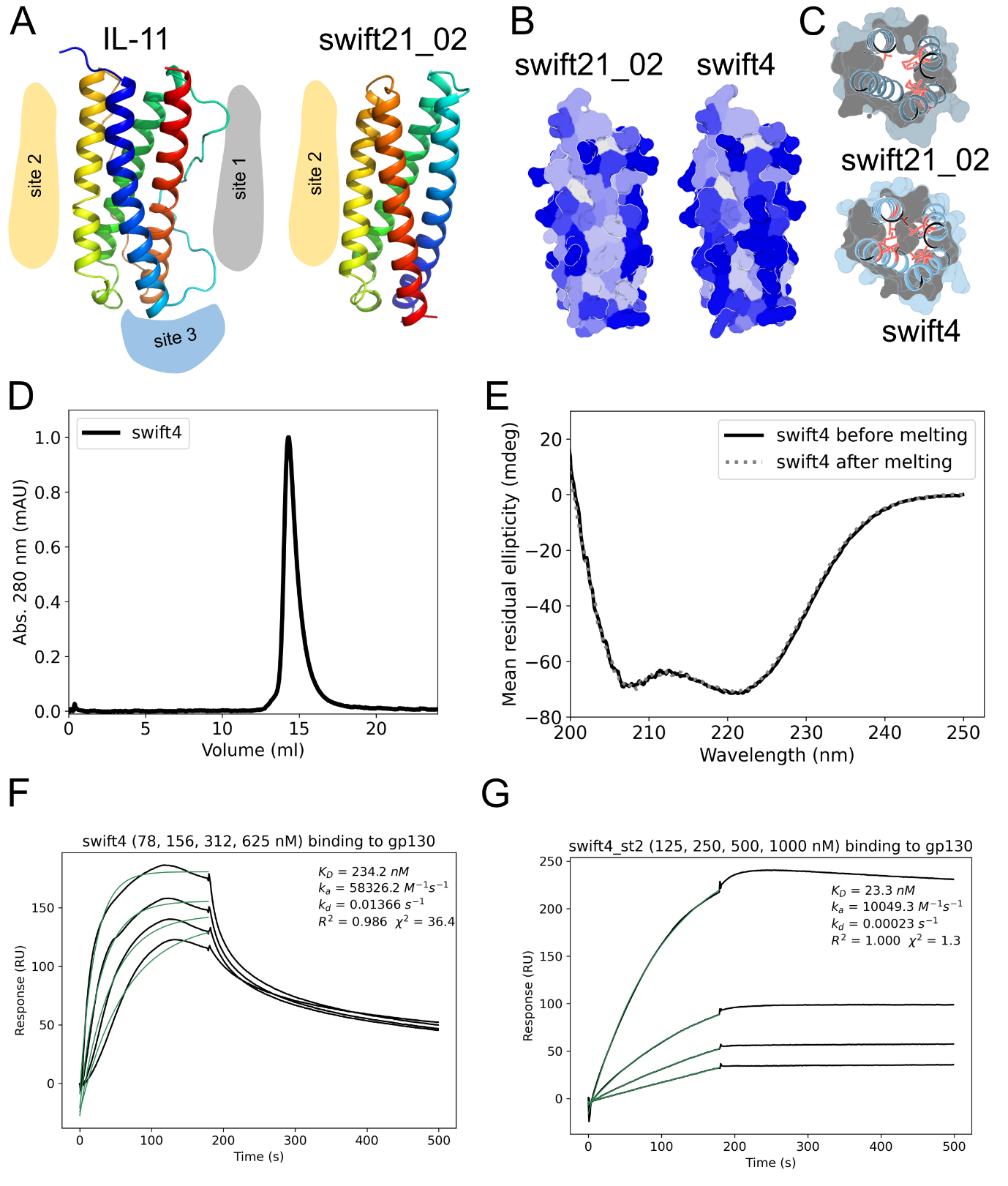


**Figure R. The design of refactored gp130 binding domains.** (A) Structure of Interleukin 11 (IL-11), an up-up-down-down helical bundle (PDB: 6O4O [6]), and the design model of swift21_02 comprising a simplified up-down-up-down that preserve one gp130 binding site (site 2). (B, C) First generation design (swift21_02) showed larger surface area of exposed hydrophobic residues (residue polarity indicated by color scale; blue: polar, white: apolar) and a poorly packed core. Surface and core residues were computationally redesigned leading to swift4. (D, E) Swift4 design was monomeric and thermostable, as analysed by SEC and CD measurements. (F, G) Swift4 binds gp130 with sub-micromolar affinity, which was 10-fold improved by tandem repetition (swift4_st2), aligning with the expected enhancement observed when comparing single and tandemly repeated domains. Details of the design and experimental procedures for the gp130 binder designs can be found in the S1 Methods. Numerical data for graphical items in this Figure can be found in S1 Data.

# ***Supplementary Tables***

**Table A.** Shown are the mutations in bv6 compared to the given design (bop1, bop2, boa1, and boa2) and the corresponding residues in G-CSF (2D9Q). The residues highlighted in red represent the additional mutations between corresponding designs with small and large secondary binding sites (e.g. bop1 and bop2).

| **bop1** | **G-CSF** | **bop2** | **G-CSF** | **boa1** | **G-CSF** | **boa2** | **G-CSF** |
| --- | --- | --- | --- | --- | --- | --- | --- |
| A5D | D104 | A5D | D104 | A9Q | Q11 | A9Q | Q11 |
| A9Y | L108 | A9Y | L108 | E10S | S12 | E10S | S12 |
| K13D | D112 | E10D | D109 | K13R | L15 | K13R | L15 |
| A16I | T115 | K13D | D112 | G14K | K16 | G14K | K16 |
| E17L | T116 | A16I | T115 | Y18M | Q20 | Y18M | Q20 |
| A20Q | Q119 | E17L | T116 | R21K | K23 | A20R | R22 |
| V101Q | Q11 | A20Q | Q119 | A98D | D104 | R21K | K23 |
| E102S | S12 | R21Q | Q120 | E102Y | L108 | A98D | D104 |
| A105R | L15 | K23E | E122 | Q106D | D112 | E102Y | L108 |
| Q106K | K16 | V101Q | Q11 | A109I | T115 | I103D | D109 |
| A109E | E19 | E102S | S12 | F110L | T116 | Q106D | D112 |
| F110M | Q20 | A105R | L15 | K113Q | Q119 | A109I | T115 |
|  |  | Q106K | K16 |  |  | F110L | T116 |
|  |  | A109E | E19 |  |  | K113Q | Q119 |
|  |  | F110M | Q20 |  |  | Q116E | E122 |
|  |  | E112R | R22 |  |  |  |  |
|  |  | I117D | D27 |  |  |  |  |

**Table B.** Sequences of constructs presented in this study. Boldface marks residues belonging to (primary: purple, secondary: black) binding sites. Italic marks residues representing additional mutations between corresponding designs with small and large secondary binding sites (e.g. bop1 and bop2).

| Construct | Sequence |
| --- | --- |
| bop1 | MAAL**D**AAL**Y**EIY**D**GL**IL**YQ**Q**RLKSLEGISPELGPAL**D**ALR**Y**DMA**D**FA**IL**MA**Q**AMEEGLDSLP**QS**FL**RK**AL**EM**IR**K**IQADAAALREKLAATYKGNDRAAAA**QS**IA**RK**LE**EM**LE**K**AYQILRHLAAA |
| bop2 | MAAL**D**AAL**Y*D***IY**D**GL**IL**YQ**Q*Q***L***E***SLEGISPELGPAL**D**ALR**Y**DMA**D**FA**IL**MA**Q**AMEEGLDSLP**QS**FL**RK**AL**EM**IR**K**IQADAAALREKLAATYKGNDRAAAA**QS**IA**RK**LE**EM**L***R*K**AYQ***D***LRHLAAA |
| bop3 | MAAL**D**AAL**Y**EIY**D**GL**IL**YQ**Q**RLKSLEGISPELGPAL**D**ALR**Y**DMA**D**FA**YL**MA**Q**AMEEGLDSLP**QR**FL**WK**AL**EM**IR**K**IQADAAALREKLAATYKGNDRAAAA**QS**IA**RK**LE**EM**LE**K**AYQILRHLAAA |
| boa1 | MAALAAAL**QS**IY**RK**LA**EM**QA**K**LKSLEGISPELGPAL**D**ALR**Y**DMA**D**FA**IL**MA**Q**AMEEGLDSLP**QS**FL**RK**AL**EM**IR**K**IQADAAALREKLAATYKGNDRA**D**AAV**Y**IAA**D**LE**IL**LE**Q**AYQILRHLAAA |
| boa2 | MAALAAAL**QS**IY**RK**LA**EM**Q***R*K**LKSLEGISPELGPAL**D**ALR**Y**DMA**D**FA**IL**MA**Q**AMEEGLDSLP**QS**FL**RK**AL**EM**IR**K**IQADAAALREKLAATYKGNDRA**D**AAV**Y*D***AA**D**LE**IL**LE**Q**AY***E***ILRHLAAA |
| bop2_st2 | MAAL**D**AAL**Y*D***IY**D**GL**IL**YQ**Q*Q***L***E***SLEGISPELGPAL**D**ALR**Y**DMA**D**FA**IL**MA**Q**AMEEGLDSLP**QS**FL**RK**AL**EM**IR**K**IQADAAALREKLAATYKGNDRAAAA**QS**IA**RK**LE**EM**L***R*K**AYQ***D***LRHLAAAGGGGSSMAAL**D**AAL**Y*D***IY**D**GL**IL**YQ**Q*Q***L***E***SLEGISPELGPAL**D**ALR**Y**DMA**D**FA**IL**MA**Q**AMEEGLDSLP**QS**FL**RK**AL**EM**IR**K**IQADAAALREKLAATYKGNDRAAAA**QS**IA**RK**LE**EM**L***R*K**AYQ***D***LRHLAAA |
| boa2_st2 | MAALAAAL**QS**IY**RK**LA**EM**Q***R*K**LKSLEGISPELGPAL**D**ALR**Y**DMA**D**FA**IL**MA**Q**AMEEGLDSLP**QS**FL**RK**AL**EM**IR**K**IQADAAALREKLAATYKGNDRA**D**AAV**Y*D***AA**D**LE**IL**LE**Q**AY***E***ILRHLAAAGGGGSSMAALAAAL**QS**IY**RK**LA**EM**Q***R*K**LKSLEGISPELGPAL**D**ALR**Y**DMA**D**FA**IL**MA**Q**AMEEGLDSLP**QS**FL**RK**AL**EM**IR**K**IQADAAALREKLAATYKGNDRA**D**AAV**Y*D***AA**D**LE**IL**LE**Q**AY***E***ILRHLAAA |
| bv21 | MAALAAALAEIYKGLAEYQARLKSLEGISPELGPAL**D**ALR**W**DMA**D**FA**YL**MA**Q**AMEEGLDSLP**QR**FL**WK**AL**EM**IR**K**IQADAAALREKLAATYKGNDRAAAAVEIAAQLEAFLEKAYQILRHLAAA |
| bv22 | MAALAAALAEIYKGLAEYQARLKSLEGISPELGPAL**D**ALR**Y**DMA**D**FA**YL**MA**Q**AMEEGLDSLP**QR**FL**WK**AL**EM**IR**K**IQADAAALREKLAATYKGNDRAAAAVEIAAQLEAFLEKAYQILRHLAAA |
| bv23 | MAALAAALAEIYKGLAEYQARLKSLEGISPELGPAL**D**ALR**Y**DMA**D**FA**YL**MA**Q**AMEEGLDSLP**QS**FL**WK**AL**EM**IR**W**IQADAAALREKLAATYKGNDRAAAAVEIAAQLEAFLEKAYQILRHLAAA |
| bv24 | MAALAAALAEIYKGLAEYQARLKSLEGISPELGPAL**D**ALR**W**DMA**D**FA**CL**MA**Q**AMEEGLDSLP**QR**FL**WK**AL**EM**IR**K**IQADAAALREKLAATYKGNDRAAAAVEIAAQLEAFLEKAYQILRHLAAA |
| Intimin_EHEC_ (1-659)-E_tag-POI-Myc_tag  (display system reading frame of pNB4) | MITHGCYTRTRHKHKLKKTLIMLSAGLGLFFYVNQNSFANGENYFKLGSDSKLLTHDSYQNRLFYTLKTGETVADLSKSQDINLSTIWSLNKHLYSSESEMMKAAPGQQIILPLKKLPFEYSALPLLGSAPLVAAGGVAGHTNKLTKMSPDVTKSNMTDDKALNYAAQQAASLGSQLQSRSLNGDYAKDTALGIAGNQASSQLQAWLQHYGTAEVNLQSGNNFDGSSLDFLLPFYDSEKMLAFGQVGARYIDSRFTANLGAGQRFFLPANMLGYNVFIDQDFSGDNTRLGIGGEYWRDYFKSSVNGYFRMSGWHESYNKKDYDERPANGFDIRFNGYLPSYPALGAKLIYEQYYGDNVALFNSDKLQSNPGAATVGVNYTPIPLVTMGIDYRHGTGNENDLLYSMQFRYQFDKSWSQQIEPQYVNELRTLSGSRYDLVQRNNNIILEYKKQDILSLNIPHDINGTEHSTQKIQLIVKSKYGLDRIVWDDSALRSQGGQIQHSGSQSAQDYQAILPAYVQGGSNIYKVTARAYDRNGNSSNNVQLTITVLSNGQVVDQVGVTDFTADKTSAKADNADTITYTATVKKNGVAQANVPVSFNIVSGTATLGANSAKTDANGKATVTLKSSTPGQVVVSAKTAEMTSALNASAVIFFDGAPVPYPDPLEPAQPA**POI**AAAEQKLISEEDAAA |
| bv6 (POI) | MAALAAALAEIYKGLAEYQARLKSLEGISPELGPAL**D**ALR**Y**DMA**D**FA**IL**MA**Q**AMEEGLDSLP**QS**FL**RK**AL**EM**IR**K**IQADAAALREKLAATYKGNDRAAAAVEIAAQLEAFLEKAYQILRHLAAA |
| bv8 (POI) | MAALAAALAEIYKGLAEYQARLKSLEGISPELGPAL**D**ALR**L**DMA**D**FA**RL**MA**Q**AMEEGLDSLP**QR**FL**YK**AL**EM**IR**K**IQADAAALREKLAATYKGNDRAAAAVEIAAQLEAFLEKAYQILRHLAAA |
| buto_a3 | LGEWKTQPEETKIKDILEAVAKLLEAVEKLRAQLGPTSLSKLLEQLAALVRLLLDALRSNGPSAEVARVLSKLLRDVHELHERLEKGLSDSEVVKAAAQIFLYFQHLLRGKLRFLML |
| buto_a3_t2 | LGEWKTQPEETKIKDILEAVAKLLEAVEKLRAQLGPTSLSKLLEQLAALVRLLLDALRSNGPSAEVARVLSKLLRDVHELHERLEKGLSDSEVVKAAAQIFLYFQHLLRGKLRFLMLGGGGSSGGGGSSGGGGSSGGGGSSLGEWKTQPEETKIKDILEAVAKLLEAVEKLRAQLGPTSLSKLLEQLAALVRLLLDALRSNGPSAEVARVLSKLLRDVHELHERLEKGLSDSEVVKAAAQIFLYFQHLLRGKLRFLML |
| swift21_02 | MSSAWAAIRAAHAILAGLHLLLDWAVRALLLLKTKSGPAEAAAVLTRLRADLLSYLRHVQWLRRAGGSSLKTLEPELATLQARLDRLLRRLQLLMSRPPSQAELDSVVLLVRSLLADTRQLAAQLRDKFP |
| swift4 | MSSAWAAIRAAHAILEGLHRLLERAVRALLRLKTKSGPEEAMKVLERLRQELAHYLRHIQAMRRAGGSSLKTLEPEFATMQARLDRLLRRLQLLMSRPPSQAELDSVVLLVRSLLADTRQLAAQVRDKFP |
| swift4_st2 | MSSAWAAIRAAHAILEGLHRLLERAVRALLRLKTKSGPEEAMKVLERLRQELAHYLRHIQAMRRAGGSSLKTLEPEFATMQARLDRLLRRLQLLMSRPPSQAELDSVVLLVRSLLADTRQLAAQVRDKFPGGGGSSMSSAWAAIRAAHAILEGLHRLLERAVRALLRLKTKSGPEEAMKVLERLRQELAHYLRHIQAMRRAGGSSLKTLEPEFATMQARLDRLLRRLQLLMSRPPSQAELDSVVLLVRSLLADTRQLAAQVRDKFP |

**Table C.** The estimated dissociation rate (*k_d_*), association rate (*k_a_*), and dissociation constant (*K_D_*) of the corresponding designs are provided the corresponding colors signify whether the samples were measured in the same experiments. SPR parameter for Boskar4 and bv6 colored in green were adopted from [4]Furthermore, the half-maximal inhibitory concentration (*IC_50_*) and the half-maximal effective concentration (*EC_50_*) of NFS-60 assays are presented, along with indications of residual activity for non-inhibiting concentrations.

| Design | SPR parameter | | | | *IC_50_* mean ± sd (nM) | *EC_50_* mean ± sd (pM) | Residual activity |
| --- | --- | --- | --- | --- | --- | --- | --- |
|  | *k_a_* (M^−1^ s^−1^) | *k_d_* (s^−1^) | *K_D_* (M) | Chi² (RU²) |  |  |  |
| bv6 | (1.2 ± 0.4) × 10^6^  (2.4 ± 0.7) × 10^5^ | (5.1 ± 1.1) × 10^-3^  (3.3 ± 0.4) × 10^-3^ | (4.3 ± 0.4) × 10^-9^  (1.4 ± 0.2) × 10^-8^ | 2.33  2.51 | 93.1 | NA | yes |
| bop1 | (8.8 ± 4.0) × 10^5^  (2.0 ± 0.6) × 10^6^ | (2.2 ± 0.6) × 10^-3^  (6.3 ± 0.7) × 10^-3^ | (2.7 ± 0.4) × 10^-9^  (3.2 ± 0.5) × 10^-9^ | 4.76  13.2 | 35.3 ± 12.9 | NA | no |
| bop2 | (1.9 ± 1.1) × 10^6^ | (1.3 ± 0.3) × 10^-3^ | (8.5 ± 3.2) × 10^-10^ | 12.7 | 58.2 ± 28.8 | 32.8 ± 9.4 | yes |
| boa1 | (6.9 ± 1.1) × 10^5^ | (2.4 ± 0.2) × 10^-3^ | (3.7 ± 0.8) × 10^-9^ | 2.18 | 106.5 ± 78.0 | NA | yes |
| boa2 | (1.4 ± 0.7) × 10^6^ | (1.3 ± 0.2) × 10^-3^ | (1.1 ± 0.4) × 10^-9^ | 12.3 | 37.8 ± 22.9 | NA | yes |
| bop3 | (1.5 ± 0.3) × 10^6^ | (1.8 ± 0.3) × 10^-3^ | (1.2 ± 0.1) × 10^-9^ | 2.1 | 13.4 ± 16.9 | NA | no |
| bop2_st2 | NA | NA | NA | NA | NA | 2.5 ± 0.6 | NA |
| boa2_st2 | NA | NA | NA | NA | NA | 12.1 ± 5.0 | NA |
| Boskar4 | (3.4 ± 1.7) × 10^4^ | (5.4 ± 1.4) × 10^-3^ | (1.7 ± 0.4) × 10^-7^ | 13.9 | 2298.2 | NA | yes |
| bv8 | (7.2 ± 6.7) × 10^5^ | (1.7 ± 1.4) × 10^-2^ | (2.6 ± 0.3) × 10^-8^ | 5.52 | 336.1 | NA | yes |
| bv21 | (8.2 ± 1.4) × 10^5^ | (1.1 ± 0.1) × 10^-3^ | (1.4 ± 0.1) × 10^-9^ | 1.34 | 47.8 | NA | yes |
| bv22 | (8.8 ± 2.2) × 10^5^  (1.4 ± 0.1) × 10^6^ | (9.2 ± 0.7) × 10^-4^  (1.8 ± 0.1) × 10^-3^ | (1.1 ± 0.2) × 10^-9^  (1.3 ± 0.1) × 10^-9^ | 3.88  3.34 | 35.0 | NA | yes |
| bv23 | (8.6 ± 1.9) × 10^5^ | (1.5 ± 0.1) × 10^-3^ | (1.8 ± 0.2) × 10^-9^ | 1.29 | 21.4 | NA | yes |
| bv24 | (1.0 ± 0.4) × 10^6^ | (1.7 ± 0.3) × 10^-3^ | (1.7 ± 0.3) × 10^-9^ | 2.86 | 108.4 | NA | Yes |

**Table D.** Crystallographic Data Collection and Refinement Statistics

| Structure | **bop1** |
| --- | --- |
| **Data collection** |  |
| Space group | P1 |
| Cell parameters  a, b, c, (*Å)* | 23.26, 29.13, 37,76, 93.02, 92.60, 97.87 |
| Wavelength (*Å)* | 1.000 |
| Resolution limits (*Å)^a^* | 23.57 – 1.30 (1.38 – 1.30) |
| Unique reflections | 41065 (4515) |
| Completeness (%) | 85.3 (58.3) |
| Redundancy | 1.79 (1.44) |
| I/σI | 7.20 (1.00) |
| R_merge_ (%) | 4.9 (53.0) |
| CC (1/2) | 99.5 (72.2) |
| **Refinement** |  |
| Resolution limits (*Å)* | 23.57 – 1.30 |
| R_cryst_ (%) | 22.6 |
| R_free_ (%) | 26.5 |
| Mean B value (*Å^2^)* | 28.551 |
| Ramachandran preferred/allowed/outlier regions (%) | 99.0/1.0/0.0 |

^a^ Values in parenthesis refer to the highest-resolution shell.

***References***

1. Abramson, J., et al., *Accurate structure prediction of biomolecular interactions with AlphaFold 3.* Nature, 2024: p. 1-3.

2. Jumper, J., et al., *Highly accurate protein structure prediction with AlphaFold.* Nature, 2021. **596**(7873): p. 583-589.

3. Skokowa, J., et al., *A topological refactoring design strategy yields highly stable granulopoietic proteins.* Nature communications, 2022. **13**(1): p. 1-17.

4. Ullrich, T., et al., *Tuning of granulopoietic signaling by de novo designed agonists.* bioRxiv, 2023: p. 2023.11. 25.568662.

5. Tsutsumi, N., et al., *Structure of the thrombopoietin-MPL receptor complex is a blueprint for biasing hematopoiesis.* Cell, 2023. **186**(19): p. 4189-4203. e22.

6. Metcalfe, R.D., et al., *The structure of the extracellular domains of human interleukin 11α receptor reveals mechanisms of cytokine engagement.* Journal of Biological Chemistry, 2020. **295**(24): p. 8285-8301.
