# Supplementary figures and images for: A strategy to design protein-based antagonists against type I cytokine receptors
Source: PLoS Biol. 2024 Nov 26;22(11):e3002883. doi: 10.1371/journal.pbio.3002883 (PMC11596305; doi:10.1371/journal.pbio.3002883)

Fig N in S1 Text

Cropped image

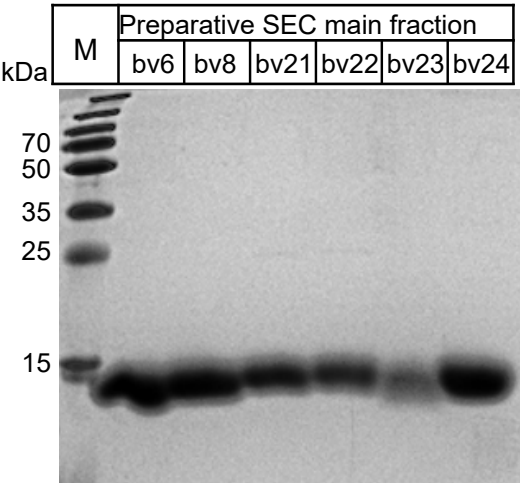

Uncropped image

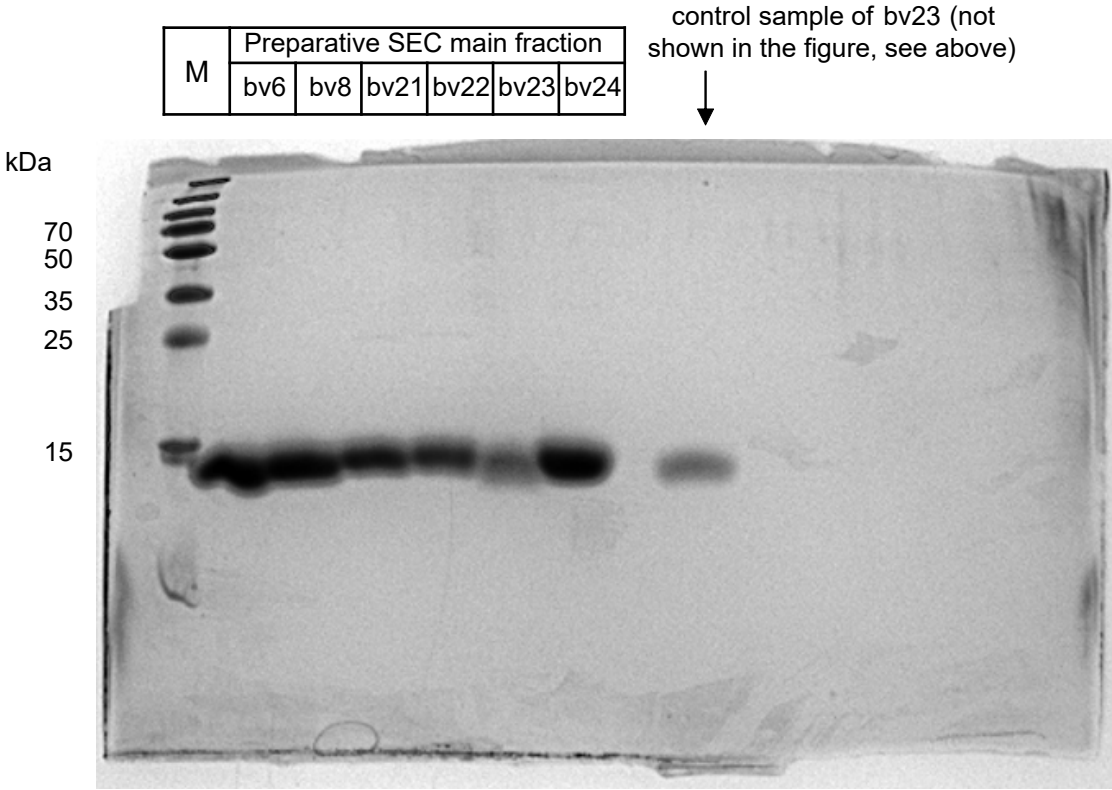

Supplement: S1 Raw Images — (PDF) [file pbio.3002883.s003.pdf]
