# Supplementary material for: A strategy to design protein-based antagonists against type I cytokine receptors
Source: PLoS Biol. 2024 Nov 26;22(11):e3002883. doi: 10.1371/journal.pbio.3002883 (PMC11596305; doi:10.1371/journal.pbio.3002883)
Supplement: S1 Methods — (DOCX) [file pbio.3002883.s004.docx]

# ***Supplementary Methods***

***Damietta input for the computationally-guided affinity maturation of bv6 and bv8***

Example Damietta specifications for weights, sample parameters, the mutable residues, and repackable residues of bv6 and bv8 for affinity maturation purposes (compare Material and methods, Computationally-guided affinity maturation).

# mutational targets

mut_res 38 ADEFHIKLMNQRSTVWY

mut_res 39 ADEFHIKLMNQRSTVWY

mut_res 42 ADEFHIKLMNQRSTVWY

mut_res 45 ADEFHIKLMNQRSTVWY

mut_res 46 ADEFHIKLMNQRSTVWY

mut_res 61 ADEFHIKLMNQRSTVWY

mut_res 64 ADEFHIKLMNQRSTVWY

mut_res 65 ADEFHIKLMNQRSTVWY

mut_res 68 ADEFHIKLMNQRSTVWY

mut_res 69 ADEFHIKLMNQRSTVWY

mut_res 72 ADEFHIKLMNQRSTVWY

# repacking targets

rpk_res 34

rpk_res 35

rpk_res 37

rpk_res 41

rpk_res 49

rpk_res 60

rpk_res 62

rpk_res 71

rpk_res 286

rpk_res 287

rpk_res 290

rpk_res 291

rpk_res 292

rpk_res 293

rpk_res 316

rpk_res 318

rpk_res 319

rpk_res 350

rpk_res 351

rpk_res 352

rpk_res 353

rpk_res 400

rpk_res 401

rpk_res 403

# sampling parameters (optional)

scramble_order 1

m_mutations 3

n_paths 7

n_iters 5

# mutagenesis scoring weights (optional)

mut_max_lj 25.0

mut_w_pp 1.0

mut_w_k 0.0

mut_w_lj 1.0

mut_w_solv 1.0

mut_w_elec 0.125

# repacking scoring weights (optional)

rpk_max_lj 25.0

rpk_w_pp 1.0

rpk_w_k 1.0

rpk_w_lj 1.0

rpk_w_solv 1.0

rpk_w_elec 0.125

***Design of TPOR binders***

To design TPOR binders, the structure of TPO (PDB: 1V7M [1]) was used as a starting template. Long loops (residues 25-49 and 109-124) were removed, the first and last helix were extended by two additional turns, and the constituting helical fragments were circularly permuted and reconnected by 3- and 4-residue loops. The core and surface residues were designed using RosettaScripts [2] protocol below, and the most conformationally-stable loop sequences were modelled using Modeller [3] and ranked by their conformational stability in molecular dynamics simulations as previously described [4].

<ROSETTASCRIPTS>

<TASKOPERATIONS>

<ReadResfile name=rrf filename=%%resfile%%/>

<IncludeCurrent name=currentTask/>

<RestrictToRepacking name=repackonly/>

<RestrictToInterfaceVector name=vectorTask chain1_num=1 chain2_num=2 CB_dist_cutoff=10.0 nearby_atom_cutoff=6.0 vector_angle_cutoff=65.0 vector_dist_cutoff=8.0/>

<InitializeFromCommandline name=cmdTask/>

</TASKOPERATIONS>

<FILTERS>

<PackStat name=holes_1 threshold=%%pck_scr1%% chain=0 repeats=5/>

<PackStat name=holes_2 threshold=%%pck_scr2%% chain=0 repeats=5/>

<ScoreType name=ttl_scr scorefxn=talaris2013 score_type=total_score threshold=%%ttl_scr_thrshld%%/>

</FILTERS>

<MOVERS>

<Backrub name=backrub/>

<BackrubDD name=backrubdd partner1=1 partner2=1 interface_distance_cutoff=8.0 moves=1000 sc_move_probability=0.1 scorefxn=talaris2013 small_move_probability=0.1 bbg_move_probability=0.1 task_operations=rrf/>

<RepackMinimize name=des1 scorefxn_repack=soft_rep scorefxn_minimize=soft_rep minimize_bb=0 task_operations=rrf design_partner1=1 design_partner2=1/>

<RepackMinimize name=des2 scorefxn_repack=talaris2013 scorefxn_minimize=talaris2013 minimize_bb=0 design_partner1=1 design_partner2=1 task_operations=rrf/>

<RepackMinimize name=des3 design_partner1=1 design_partner2=1 minimize_bb=1 task_operations=rrf/>

<FastRelax name=relax scorefxn=talaris2013 repeats=2 task_operations=currentTask,repackonly,cmdTask/>

<ParsedProtocol name=design>

<Add mover_name=des1/>

<Add mover_name=backrub/>

<Add mover_name=des1/>

<Add mover_name=des2/>

<Add mover_name=des2/>

<Add mover_name=backrubdd/>

<Add mover_name=des3/>

<Add filter=holes_1/>

<Add mover_name=relax/>

</ParsedProtocol>

<GenericMonteCarlo name=iterate filter_name=ttl_scr scorefxn_name=talaris2013 mover_name=design trials=3/>

<GenericMonteCarlo name=iterate_h filter_name=holes_1 scorefxn_name=talaris2013 mover_name=design trials=2/>

</MOVERS>

<PROTOCOLS>

<Add mover=iterate/>

<Add filter=holes_1/>

<Add mover=iterate_h/>

<Add filter=holes_2/>

<Add filter=ttl_scr/>

</PROTOCOLS>

</ROSETTASCRIPTS>

The above 400 instances of the above protocol were executed as follows:

for fn in *.pdb; do for e in `seq 01 400`; do qsub -l h_vmem=3G -l h_rt=20:0:0 -cwd -N des_p1 -o ./ -b y ~/rstta_bin/rosetta_scripts.linuxgccrelease -database ~/rstta_db -s $fn -docking:dock_pert 3 2 -nstruct 100 -out:prefix $e -out:file:silent slnt_$e -out:file:scorefile score_$e -parser:protocol core_redes_fixbb_ttl_nrgy_09.xml -parser:script_vars resfile='csf_resfile' pck_scr1='0.50' pck_scr2='0.53' ttl_scr_thrshld='-210.0' -mute all -ex1 -ex2; done; done

***Bacterial protein expression and purification of TPOR binders***

Synthetic genes encoding the human TPO (Ser22 – Ser184) and the designs (Table S2) were cloned into the pET28a(+) expression vector between the NdeI and XhoI cloning sites in-frame with a thrombin cleavage site and an N-terminal poly-histidine purification tag (Synbio Technologies, Inc.). Plasmids were transformed into chemically competent E. coli BL21(DE3) using the heat shock method. Transformed cells were grown in LB medium supplemented with 40 μg/ml kanamycin at 37 °C. At OD600 of 0.6-1.0, cells were induced with 1mM IPTG and incubated overnight at 25°C for protein expression. Cells were harvested by centrifugation at 5000 g at 4°C for 20 min and lysed in 25 ml of lysis buffer (1M guanidinium chloride, 100 mM NaCl, 50mM Tris-HCl pH 8.0) supplemented with a tablet of the cOmplete, EDTA-free Protease Inhibitor Cocktail (Roche, 5056489001) and 3 mg of lyophilized DNase I (PanReac AppliChem, A3778) using a Branson Sonifier S-250 (Fisher Scientific). The lysate was cleared by centrifugation at 28000 g for 50 min and the supernatant was passed through a 0.45 μm filter (Millipore, SLHV033RS). The sample was applied to a 5 ml HisTrap HP column (Cytiva, GE17-5248-01). The running buffer was 150 mM NaCl, 30 mM Tris-HCl pH 8.0. After sequential washing the column with 30 ml of the running buffer supplemented with 0 or 50 mM imidazole, fractions were collected by linear gradient elution using 150 mM NaCl, 30 mM Tris-HCl pH 8.0, 500 mM imidazole buffer. The eluted fractions containing the protein of interest were pooled, concentrated using 10 kDa MWCO centrifugal filters (Millipore, UFC901024), and further purified on a Superdex Increase 75 10/300 gel filtration column (Cytiva, 29148721) using PBS. Gel filtration fractions containing pure protein in monomeric state were pooled, concentrated, and stored at -20 °C for subsequent analyses. Both IMAC and gel filtration steps were performed on an Äkta Pure chromatography system (Cytiva).

***Surface plasmon resonance (SPR) assay for TPOR binders***

Multi-cycle kinetics experiments were performed on a Biacore X100 system (GE Healthcare Life Sciences). Recombinant human TPOR (R&D Systems, 4444-TR-050) was diluted to 50 μg/mL in 10 mM acetate buffer pH 5.0 and immobilized on the surface of a CM5 sensor chip (Cytiva, 29149604) using standard amine coupling chemistry. Immobilization level was ~2000 RU. TPO, buto_a3, or buto_a3_t2 were diluted in running buffer (PBS pH 7.4 with 0.05% Tween20). Analyses were conducted at 25°C at a flow rate of 30 μL/min. Four concentrations of the sample solution (for TPO: 1.7, 5.8, 8.7, 13.0 μM; for buto_a3: 1.5, 3.1, 6.3, 25 μM; for buto_a3_t2: 0.7, 1.0, 2.2, 3.3 μM) were injected over the functionalized sensor chip surface for 180 s, followed by a 180 s dissociation with running buffer. At the end of each run, the sensor surface was regenerated with a 60 s injection of 10 mM glycine-HCl pH 2.0. The reference responses and zero-concentration sensograms were subtracted from each dataset (double-referencing). The equilibrium dissociation constants (*K_d_*) were calculated from a plot of steady state binding levels against analyte concentrations using a 1:1 interaction model. To estimate the reliability of the fit, the fitting procedure was repeated excluding one concentration at a time. This gave average *K_d_* values and standard deviations for a single titration series.

***Design of gp130 binders***

The structure of IL-11 (PDB: 6O4O [5]) was used as template for design of gp130 binders, where the long loop segments (residues 45-70 and 126-145) were removed, and the sequence was circularly permuted to introduce four- and three-residue loops instead. The sequence optimization of the designed structure was initially performed with the same RosettaScritpts protocol described above for the TPOR binders. However, upon experimental characterization of two designs, the well-expressed design (swift21_02) was not sufficiently stable for *in vitro* characterization. Therefore, we performed another round of computational design using swift21_02 as a template. This design round aimed to optimize several solvent-exposed hydrophobic residues as well as regions of the core which were not well-packed. In this iteration the Damietta (v1.07 [6, 7]) combinatorial sampler was used with the following input spec file (provided below), where 100 instances randomized order design simulations were performed, excluding wild-type residues at exposed positions. These simulations lead to 210 unique sequences, which were subsequently ranked by their conformational stability in molecular simulations where two candidates were selected for experimental characterization (swift3, and swift4; Table S2). The following spec file was generated used for the design, where the surface positions were selected according to high probability predictions of ProteinMPNN [8] (using model weights v_48_020, T=0.1, and seed=37):

library /path/to/damietta_v099_linux_x86_64/libv099_100

input swift_mut_autopsf.pdb

mut_res 10 AFILMVWY

mut_res 14 AFILMVWY

mut_res 17 AFILMVWY

mut_res 58 AFILMVWY

mut_res 61 AFILMVWY

mut_res 76 AFILMVWY

mut_res 79 AFILMVWY

mut_res 120 AFILMVWY

mut_res 124 AFILMVWY

mut_res 15 ASED

mut_res 19 GERHSDTQAK

mut_res 22 E

mut_res 23 RENSDA

mut_res 30 ERSDQAL

mut_res 38 ESDQA

mut_res 41 RLQAM

mut_res 42 ERSQATK

mut_res 45 GRESQAT

mut_res 49 GERSDQT

mut_res 50 E

mut_res 52 QAE

mut_res 53 VGREHSDTQA

mut_res 60 GERNHSDQAT

# repacking targets

rpk_res 13

rpk_res 18

rpk_res 20

rpk_res 21

rpk_res 26

rpk_res 32

rpk_res 33

rpk_res 34

rpk_res 39

rpk_res 46

rpk_res 48

rpk_res 51

rpk_res 54

rpk_res 55

rpk_res 56

rpk_res 57

rpk_res 62

rpk_res 63

rpk_res 69

rpk_res 72

rpk_res 75

rpk_res 80

rpk_res 83

rpk_res 87

rpk_res 94

rpk_res 113

rpk_res 114

rpk_res 117

rpk_res 118

rpk_res 123

scramble_order 1

m_mutations 3

n_paths 8

n_iters 3

***Bacterial protein expression and purification of gp130 binders***

Plasmids with the pET-28a(+) backbone harbouring the designs genes between NdeI and XhoI were transformed into chemi-competent *E. coli* by heat shock. Cells were grown at 37 °C and 180rpm in LB supplemented with 50 µg/mL kanamycin to an optical density at 600 nm (OD_600_) between
0.7 and 0.9 before induction of protein expression using isopropyl _β_-D-1-thiogalactopyranoside (IPTG) in a final concentration of 1 mM, at 25 °C and 180 rpm for 18 hours. The cells were harvested by centrifugation at 6000g and 8 °C for 15min, the supernatant was discarded and the pellet frozen in liquid nitrogen and stored at −80^°^C or directly used for purification. The pellet was lysed in lysis buffer (20 mM Tris pH 7.4, 500 mM NaCl, 4 mM MgCl_2_, 20 µg/mL DNase I (#A3778, ITW reagents), cOmplete^™^ EDTA-free protease inhibitor cocktail (#04693132001, Roche) and 1mM PMSF) using a precooled french pressure cell press at 1000 psi and four cycles. The lysate was centrifuged at 16000g and 8^°^C for 45min and the supernatant was used for nickel immobilized metal affinity chromatography (IMAC). The supernatant was clarified using a 0*.*45 µm syringe filter and loaded onto a Ni-NTA HisTrap column (GE Healthcare), equilibrated with 10 % IMAC buffer B (IMAC buffer A: 20 mM Tris pH 8, 500 mM NaCl; IMAC buffer B: 20 mM Tris pH 8, 500 mM NaCl, 500 mM imidazole). The column was washed for 30 CV with 10 % IMAC buffer B and eluted using a linear gradient elution from 10 to 100 % IMAC buffer B over 20CV. The protein was concentrated using Amicon Ultra Centrifugal Filters (10 kDa MWCO, #UFC9010, Millipore) and filtered through a 0*.*22 µm syringe filter. Pooled and concentrated fractions were then further purified by size exclusion chromatography (SEC) using Superdex 75 (#28989333, #28989334 and #17517401, Cytiva) columns equilibrated with phosphate-buffered saline (PBS) high salt (10 mM Na_2_HPO_4_, 1*.*8 mM KH_2_PO_4_, 300 mM NaCl, 2*.*7 mM KCl, pH 7.4). Fractions presumably containing the correct protein were pooled, concentrated using Amicon Ultra Centrifugal Filters, sterile filtered using 0*.*22 µm syringe filters, flash frozen in liquid nitrogen and stored at −80 ^◦^C. Analytical SEC was performed by injecting ∼500 µg of protein onto a Superdex 75 10/300 GL column (#17517401, Cytiva) using a 100 µL capillary loop, with a flow rate of 0*.*8 mL/min. PBS high salt was used as running buffer.

***Melting analysis with circular dichroism (CD) for gp130 binders***

Circular dichroism (CD) spectra were measured using an JASCO J-810 spectropolarimeter with 400 µL sample diluted to ∼0*.*2 mg/mL in CD buffer (10 mM KH_2_PO_4_, 100 mM KCl, pH 7.4) in 1mm path length cuvettes. Spectra were constructed from four accumulated measurements over 195 to 250 nm, with a data pitch of 0*.*1nm, a bandwidth of 1nm, a response of 1s and a scanning speed of 20 nm/min at 20 °C. A blank spectrum was recorded for every protein by diluting the respective buffer in CD buffer. To measure CD melting curves, a temperature gradient of 1 °C/min from 20 to 95 °C was applied to the sample and the ellipticity *θ* at 222 nm was measured every 0*.*5 ^◦^C. Additionally, a full spectrum from 195 to 250nm with five accumulations and a data pitch of 0*.*1nm, a bandwidth of 1nm, a response of 1s and a scanning speed of 100 nm/min was recorded every 10 °C.

***Surface plasmon resonance (SPR) assay for gp130 binders***

Surface plasmon resonance (SPR) multi-cycle kinetic experiments were performed using a Biacore X100 system (GE Healthcare) to determine binding kinetics. Carrier free recombinant human gp130 Fc Chimera Avi-tag protein (#AVI10929, R&D Systems) was diluted to 100µgmL^−1^ in HBS-EP^+^ buffer (10mM HEPES, 150mM NaCl, 3mM EDTA, 0*.*05% Tween-20, pH 7.4) and immobilized on a sensor chip SA (#BR100398, Cytiva) using the non-covalent biotin-streptavidin interaction. In detail, the sensor surface was conditioned by three consecutive one-minute injections of 1 M NaCl in 50mM NaOH, and the biotinylated ligand was immobilized to the sensor surface of flow cell 2 with a 1min contact time. After immobilization, the system was washed using 50% isopropanol in 1 M NaCl and 50mM NaOH. The final immobilization level reached was 2499RU and all measurements of this thesis were performed using the same sensor chip. Protein samples were centrifuged at 17000g and 8 ^◦^C for 10min to remove aggregates before being diluted in HBS-EP^+^, and multi-cycle kinetics were measured at 25 ^◦^C using HBS-EP^+^ as the running buffer. Samples were injected from the lowest to the highest concentration for 180s, followed by a 360s dissociation phase and regeneration of the surface for 60s using 10mM glycine-HCl. The reference cell (flow cell 1) response was subtracted from the measurement cell (flow cell 2) response, additionally the response of a blank injection was subtracted. The association rate constant (*k*_a_), dissociation rate constant (*k*_d_) and equilibrium dissociation constant (*K*_D_) were obtained by the *k*_obs_ linearization model using a 1:1 binding model [4, 9].

# ***References***

1. Feese, M.D., et al., *Structure of the receptor-binding domain of human thrombopoietin determined by complexation with a neutralizing antibody fragment.* Proceedings of the National Academy of Sciences, 2004. **101**(7): p. 1816-1821.

2. Fleishman, S.J., et al., *RosettaScripts: a scripting language interface to the Rosetta macromolecular modeling suite.* PLoS One, 2011. **6**(6): p. e20161.

3. Eswar, N., et al., *Comparative protein structure modeling using Modeller.* Curr Protoc Bioinformatics, 2006. **Chapter 5**: p. Unit-5.6.

4. Skokowa, J., et al., *A topological refactoring design strategy yields highly stable granulopoietic proteins.* Nature Communications, 2022. **13**(1): p. 2948.

5. Metcalfe, R.D., et al., *The structure of the extracellular domains of human interleukin 11α receptor reveals mechanisms of cytokine engagement.* Journal of Biological Chemistry, 2020. **295**(24): p. 8285-8301.

6. Maksymenko, K., et al., *The design of functional proteins using tensorized energy calculations.* Cell Reports Methods, 2023. **3**(8): p. 100560.

7. Grin, I., et al., *The Damietta Server: a comprehensive protein design toolkit.* Nucleic Acids Research, 2024. **52**(W1): p. W200-W206.

8. Dauparas, J., et al., *Robust deep learning–based protein sequence design using ProteinMPNN.* Science, 2022. **378**(6615): p. 49-56.

9. Krämer, S.D., et al., *Anabel: An Online Tool for the Real-Time Kinetic Analysis of Binding Events.* Bioinform Biol Insights, 2019. **13**: p. 1177932218821383.
